# A longitudinal study on determinants of HPV vaccination uptake in parents/guardians from different ethnic backgrounds in Amsterdam, the Netherlands

**DOI:** 10.1186/s12889-017-4091-4

**Published:** 2017-02-21

**Authors:** Catharina J. Alberts, Maarten F. Schim van der Loeff, Yvonne Hazeveld, Hester E. de Melker, Marcel F. van der Wal, Astrid Nielen, Fatima El Fakiri, Maria Prins, Theo G. W. M. Paulussen

**Affiliations:** 1 Department of Infectious Diseases, Research and Prevention, Public Health Service (GGD) of Amsterdam, Nieuwe Achtergracht 100, 1018 WT Amsterdam, The Netherlands; 2 Department of Internal Medicine, Division of Infectious Diseases, Center for Infection and Immunity Amsterdam (CINIMA), Academic Medical Center (AMC), Amsterdam, The Netherlands; 3 Department of Youth Health Service, Public Health Service of Amsterdam (GGD), Amsterdam, The Netherlands; 4 Department of Epidemiology and Surveillance, National Institute of Public Health and the Environment (RIVM), Bilthoven, The Netherlands; 5 Department of Epidemiology and Health Promotion, Public Health Service of Amsterdam (GGD), Amsterdam, The Netherlands; 6 TNO (Netherlands Organization for Applied Scientific Research), Expertise Centre Child Health, Leiden, The Netherlands

**Keywords:** HPV, Vaccination acceptability, Vaccination uptake, Population-based, Ethnicity, the Netherlands

## Abstract

**Background:**

Human papillomavirus (HPV) vaccination coverage in the Netherlands is low (~60%) compared to other childhood vaccinations (>90%), and even lower among ethnic minorities. The aim of this study was to explore the possible impact of ethnicity on the determinants of both HPV vaccination intention and HPV vaccination uptake among parents/guardians having a daughter that is invited for the HPV vaccination.

**Methods:**

In February 2014, parents/guardians living in Amsterdam were invited to complete a questionnaire about social-psychological determinants of their decision making process regarding the HPV vaccination of their daughter and socio-demographic characteristics. This questionnaire was sent approximately one month before the daughter was scheduled to receive her first HPV vaccine dose. Their daughters’ HPV vaccination status was retrieved from the national vaccination database. We distinguished four ethnic groups: Dutch (NL), Surinamese, Netherlands Antillean, and Aruban (SNA), Middle-Eastern and North-African (MENA), and Other. To assess the impact of determinants on both intention and uptake, linear and logistic regression analyses were used respectively. Missing data were imputed using multiple imputation by chained equation.

**Results:**

In total 1,309 parents/guardians participated (33% participation rate). In all groups we found the mothers’ intention to be the strongest predictor of their daughters’ HPV vaccination uptake. Explained variance of uptake was highest in the NL-group (pseudo-R^2^:0.56) and lower in the other ethnic groups (pseudo-R^2^ varied between 0.23 and 0.29). The lower explained variance can be attributed to the relative large proportion of participants with a positive intention that finally did not go for vaccination in the SNA-group (11%) and MENA-group (30%). Explained variance (R^2^) of intention varied between 0.66 and 0.77 across ethnic groups, and was best explained by the proximal social-psychological determinants. The strength of association of these determinants with both intention and uptake were largely similar across ethnic groups.

**Conclusion:**

We conclude that the same determinants should be targeted in the different ethnic groups, although the mode of delivery of the intervention needs to be tailored to the different cultural backgrounds. Further research is needed to explain the observed discrepancy between intention and uptake, especially among parents/guardians in the non-Dutch groups.

**Electronic supplementary material:**

The online version of this article (doi:10.1186/s12889-017-4091-4) contains supplementary material, which is available to authorized users.

## Background

The national immunization program (NIP) of the Netherlands is a voluntary and free-of-charge program with an overall high childhood vaccination uptake that exceeds 90% [1]. However, in 2009 when the HPV vaccination was added to the NIP, the uptake of this particular vaccination was drastically lower: 52% in 2009 [2], increasing to 61% in 2014 [1]. Lower uptake has been observed in certain ethnic minorities; e.g. in 2009 an uptake of 44%, 38%, and 24% was observed among girls of which both parents were born in Surinam, Turkey, or Morocco, respectively [3]. This is especially worrying as these groups are known to have a higher incidence of cervical cancer compared to the native Dutch population [4]. Similar ethnic disparities are observed around the globe [5–9].

In previous studies investigating HPV vaccination acceptability in the Netherlands, common predictors of HPV vaccination intention appeared to be attitude, concerns about vaccination effectiveness and safety, feelings of ambivalence towards HPV vaccination, anticipated regret, and normative beliefs [7, 10, 11]. Factors that did not appear to have an effect on HPV vaccination acceptability were knowledge and knowing someone with an abnormal Pap test [10, 12–14]. Studies focussing on HPV vaccination acceptability among ethnic minorities are mostly small and/or struggle with answering why differences in uptake between ethnicities exist [9, 11, 15, 16]. The aim of this study is to explore the process of decision making by parents/guardians from different ethnic backgrounds about the HPV vaccination uptake of their daughter. The following research questions were central in this study: (1) What are the determinants in the process of parental decision making about the HPV vaccination of their daughter?, and (2) Do these determinants differ between parents/guardians from different ethnic backgrounds?

## Methods

In the Netherlands the bivalent HPV vaccine (Cervarix) is offered since 2009 by the National Immunization Programme (NIP). In 2014, the vaccination schedule was reduced from three to two doses in line with guidelines of the European Medicine Agency. All girls are invited for HPV vaccination in the year they turn 13 years old. Girls are invited to come to a large public venue designated by the Public Health Service (e.g. sports centers and child-and parent centers). During this vaccination moment no personal consultation is offered and no consent of the parent is necessary to obtain the HPV vaccination.

### Participants

All parent/guardians with a daughter born in 2001 living in the district of the Youth Health Service of the Public Health Service of Amsterdam were invited for this study (2014). They were selected from a database of the Youth Health Service of the Public Health Service of Amsterdam which contains the most up to date information of all girls residing in Amsterdam. The selection of the girls eligible for this study was done one month before they received the invitation of the HPV vaccination. Parents/guardians were able to participate only once. A computer tablet was raffled among participants. The study was approved by the Ethics Committee of the Academic Medical Centre (W2013_257).

### Invitation procedure

Parents/guardians were invited to complete a questionnaire approximately one month before their daughter was scheduled to receive her first HPV vaccine dose (see Additional file 1: Figure S1 for key dates of the recruitment period). Participants provided informed consent for this study by completing and returning the questionnaire. Since our primary aim was to predict the outcome of their decision making regarding the HPV vaccination of their daughter, we restricted our analyses to parents/guardians who returned the questionnaire before their scheduled vaccination day (see Additional file 1: Figure S2 for a flow diagram).

The questionnaire could be completed online or on paper and was available in three languages Dutch, Turkish, and English. All parents/guardians received a Dutch version of the questionnaire, and based on the country of birth of one of the parents they also received an English or a Turkish version.

In order to increase the response rate among our target population we formed a team of research assistants fluent in Dutch and at least one other relevant language (i.e. Turkish, Berber, Arabic, English, Spanish, or Twi (a local language in Ghana)). Based on the country of birth of the parents/guardians, the research assistants were matched to the language presumably spoken by the parents/guardians. The assistants attended a comprehensive training in which they were instructed about: (i) the general background of the HPV vaccination, (ii) what information they were allowed to provide to the parents/guardians in order to not influence parents’/guardians’ responses to the questionnaire, (iii) the content of the questionnaire, and (iv) relevant other logistic aspects concerning data collection and the HPV vaccination of their daughter. The assistants called parents/guardians to explain the rationale behind the study, to offer assistance with completing the questionnaire if necessary, and/or to remind them in case of an initial non-response. If parents asked information regarding the HPV vaccination, the research assistants only gave the information that was provided in the invitation letter for the HPV vaccination and the information leaflet of this study (in practice, most questions concerned location and time of vaccination and concerns regarding the safety of the HPV vaccine).

### Questionnaire

The questionnaire was based on a questionnaire initially developed by van Keulen et al*.* [10] which was successfully applied in a study on HPV vaccination intention among parents/guardians with a native Dutch background. Adaptations were based on one-on-one interviews and focus groups with native Dutch, Moroccan, Turkish, Ghanaian, and Surinamese parents, separately. The Reasoned Action approach [17], Social Cognitive Theory [18], and the Health Belief Model [19] constituted the theoretical framework of this study. The adapted questionnaire was pretested among parents/guardians and subsequently revised.

### Ethnic group

The ethnic background of participants was based on country of birth of both parents as described previously [20]. In short, a girl was classified as non-Dutch if at least one parent was born outside the Netherlands. If the mother and the father did not have the same country of birth, the country of birth of the mother was decisive. Based on their ethnicity an individual was categorized into one of the following ethnic groups; (i) Dutch (NL), (ii) Surinamese, Netherlands Antillean, and Aruban (SNA), (iii) Middle Eastern and North African (MENA) (including Turkish), and (iv) other. The MENA was grouped by the region grouping of the WHO (except for Turkey) [21] and was mainly composed of individuals with a Moroccan (*n* = 109), Turkish (*n* = 67), and Egyptian (*n* = 21) ethnicity. The category Other consisted mainly of Indonesians (*n* = 35), Ghanaians (*n* = 32), and Germans (*n* = 15).

### HPV and childhood vaccination intention and uptake

Consent to retrieve the vaccination status of the daughter was obtained by posing a separate question asking whether the parent/guardian agreed if we would retrieve the HPV and childhood vaccination status of their daughter from Praeventis. Praeventis is a national database for monitoring childhood vaccinations in the Netherlands [22]. HPV vaccination status was dichotomized: (i) Received one or both HPV vaccinations, versus (ii) Received no HPV vaccination. Intention was assessed by a composite score of two 5-point Likert-type items (Cronbach α = 0.96).

### Determinants of HPV vaccination intention and uptake

According to their theoretically expected cause-effect relation, socio-psychological determinants of HPV vaccination intention and HPV vaccination uptake were subdivided into *proximal determinants* versus *distal determinants*.

*Proximal determinants* are theoretically expected to impact the parents’/guardians’ intention most directly. These were: general attitude, vaccination-related beliefs, negative outcome expectancies, risk perception (e.g. perceived susceptibility when not vaccinated against HPV), anticipated regret, perceived relative effectiveness of the HPV vaccination, subjective norms, descriptive norm, and self-efficacy.

The impact of *distal determinants* on the parents’/guardians’ intention are expected to be indirect. These were: knowledge, confidence in authorities, ambivalence about the HPV vaccination, habit strength, information processing, amount of information processed, subjective evaluation of the received information about the HPV vaccination, past experience with cervical cancer (self or close relative), and socio-demographic characteristics. Socio-demographics included age, gender, educational level, religion (no religion, Protestantism, Catholicism, Islam, and other), and ethnicity. For analyses religion was dichotomised into religious versus non-religious.

See Additional file 1: Table S1 for more details about the items used per construct, response format, and the internal consistency of composite variables. In short, all socio-psychological determinants were assessed by a composite score (except for risk perception and anticipated regret). Composite scores were calculated when the items showed sufficient internal consistency (Chronbach’s α > 0.60). The scores were computed by summing all the items of the construct and dividing the sum by the number of items. Scores were rescaled from −2 to 2, where appropriate.

### Statistical methods

Response rate was explored by country of birth of the mother of the girl invited for the HPV vaccination; the country of birth of the mother was grouped into the same four groups as used for ethnicity of the participant. We compared HPV vaccination status, HPV vaccination intention, past childhood vaccination status, and socio-demographics by ethnicity. Descriptive analyses were only computed for complete cases.

Bivariable and multivariable analyses were based on multiple imputed datasets. We used multiple imputation to account for missing data [23]. The percentage of missing values per variable varied between <1 and 7%, except for subjective norm, for which 33% of participants did not answer at least one of the individual items. Most unanswered questions were presumably not answered because of the length of the questionnaire, and participants seemed to skip questions that appeared similar to them. We observed that participants with a lower intention to vaccinate and/or with a non-Dutch ethnicity had more data missing, compared to participants with a high intention and/or Dutch ethnicity. We used multiple imputation to create 15 multiple imputed datasets. Model parameters were combined using Rubin’s rule, combined estimates are presented in the tables [24].

HPV vaccination uptake and HPV vaccination intention were both used as criterion variable (i.e. outcome variable). Determinants of these two criterion variables, HPV vaccination intention and HPV vaccination uptake, were investigated using linear and logistic regression analyses, respectively. The multivariable analyses with uptake as the criterion variable were executed in three steps, by consecutively adding the following three sets of determinants: intention, proximal determinants, and distal determinants. Determinants were only included if the bivariable association with the criterion variable was *p* < 0.25. To obtain a parsimonious model, backward selection was executed during each step in which a new set of determinants was offered to the model (*p* < 0.05). Multivariable analyses with HPV vaccination intention as the criterion variable were executed according to the same theory-based order. In both final multivariable models we tested for interaction effects between ethnic group and each determinant (*p* < 0.05). Statistical analyses were performed in Stata 14 [25].

## Results

### Response rate

Parents/guardians of 4,216 girls were invited to participate in this study. The response rate was highest among mothers born in the Netherlands (37%), followed by the Other group (34%), SNA group (31%) and with the lowest response rate among mothers born in one of the Middle East or North African countries (20%) (see Additional file 1: Table S2 for more detail).

Of all parents/guardians invited, 1,362 (33%) returned a questionnaire of whom 1,317 (96%) returned the questionnaire before the first scheduled HPV vaccination day. Ethnicity was missing for 8 participating parents/guardians. This resulted in a net-response of 1,309 participants (see Additional file 1: Figure S2 for details). HPV vaccination uptake among these 1,309 participants was higher when compared to the overall uptake in Amsterdam in 2014 (76% in complete case analyses, 73% in the imputed dataset, and 51% in the total invited population, see Additional file 1: Figure S3).

### Baseline characteristics and differences between ethnic groups in complete cases

The majority of participants were mothers/female guardians (88%), median age was 45 years (inter quartile range (IQR): 42–48), and 28% received higher education. On average, the NL-group was oldest (median 46 (IQR: 43–49)) and reported the highest level of education (34%), 74% of the NL-group reported not to be religious, while in the MENA-group 80% reported to be Islamic, and, finally in the NL-group only 7% received help when completing the questionnaire while this was 43% in the MENA-group (see Table 1).

**Table 1:** Baseline socio-demographic characteristics, HPV vaccination intention, actual childhood vaccination and HPV vaccination uptake, by ethnic group of the parents/guardians, HPV vaccination acceptability study in Amsterdam, the Netherlands, 2014

Region of origin														
	NL		SNA			MENA			Other			Total		
	(*n* = 723)		(*n* = 126)			(*n* = 237)			(*n* = 223)			(*n* = 1309)		
*Socio-demographic characteristics*	n	%	n	%	*p vs NL*	n	%	*p vs NL*	n	%	*p vs NL*	n	%	*p overall*
Gender														
Female	630	90%	108	89%	0.905	180	82%	**0.002**	190	89%	0.863	1108	88%	**0.017**
Male	73	10%	13	11%		40	18%		23	11%		149	12%	
Age														
Median (IQR)	46	(43–49)	43	(39–47)	**<0.001**	42	(37–47)	**<0.001**	45	(41–48)	**<0.001**	45	(42–48)	**<0.001**
≤ 43 year	203	29%	61	51%	**<0.001**	119	58%	**<0.001**	86	42%	**0.004**	469	38%	**<0.001**
44–47 year	253	36%	32	27%		41	20%		63	30%		389	32%	
≥ 48 year	238	34%	27	23%		45	22%		58	28%		368	30%	
Education^a^														
Low	204	29%	71	60%	**<0.001**	137	65%	**<0.001**	80	38%	**0.033**	492	40%	**<0.001**
Intermediate	255	37%	34	29%		43	20%		61	29%		393	32%	
High	236	34%	13	11%		32	15%		67	32%		348	28%	
Religion														
No religion	515	74%	19	16%	**<0.001**	11	5%	**<0.001**	94	46%	**<0.001**	639	52%	**<0.001**
Protestantism^b^	105	15%	50	42%		13	6%		41	20%		209	17%	
Catholicism^c^	67	10%	17	14%		19	9%		40	20%		143	12%	
Islam	2	0%	16	13%		168	80%		13	6%		199	16%	
Other	6	1%	17	14%		0	0%		16	8%		39	3%	
Language used to complete the questionnaire														
Dutch	723	100%	126	100%	N.A.	194	82%	**<0.001**	199	89%	**<0.001**	1242	95%	**<0.001**
Another language than Dutch^d^	0	0%	0	0%		43	18%		24	11%		67	5%	
Received help to complete the questionnaire														
Did not receive help	649	93%	104	86%	**0.011**	124	57%	**<0.001**	160	75%	**<0.001**	1037	83%	**<0.001**
Received help^e^	50	7%	17	14%		94	43%		54	25%		215	17%	
Way of completing the questionnaire														
On paper	401	55%	70	56%	0.985	158	67%	**0.002**	137	61%	0.115	766	59%	**0.015**
Online	322	45%	56	44%		79	33%		86	39%		543	41%	
Intention (5 point-scale, range −2 to 2)														
Mean (SD)	1.30	1.17	1.33	0.92	0.850	0.68	1.30	**<0.001**	1.31	0.97	0.903	1.20	1.17	**<0.001**
	n	%	n	%		n	%		n	%		n	%	
HPV vaccination status														
No vaccination	84	13%	16	16%	**<0.001**	72	41%	**<0.001**	33	17%	**0.031**	205	18%	**<0.001**
One vaccination	22	3%	13	13%		13	7%		13	7%		61	5%	
Two vaccinations	539	84%	71	71%		92	52%		146	76%		848	76%	
Infant, toddler and preschool children vaccination^f^														
Not all	13	2%	7	7%	**0.004**	5	3%	0.515	17	9%	**<0.001**	42	4%	**<0.001**
All	632	98%	93	93%		172	97%		175	91%		1072	96%	
School-age vaccination^g^														
Not all	24	4%	5	5%	0.538	6	3%	0.835	14	7%	**0.031**	49	4%	0.169
All	621	96%	95	95%		171	97%		178	93%		1065	96%	
Overall childhood vaccination status^h^														
Not all	24	4%	8	8%	0.050	8	5%	0.626	20	10%	**<0.001**	60	5%	**0.002**
All	621	96%	92	92%		169	95%		172	90%		1054	95%	

* *p*-values of categorical variables are based on Pearson’s chi-square test for categorical variables, *p*-values for continuous variables when comparing one of the “region of origin” categories with the Dutch based on the *t*-test p-values for continuous variables overall are based on the Kruskall-Wallis test. For calculation of the *p*-values missing categories were excluded

^a^Education was based on the highest level of education and categorized into low (no education, primary school), intermediate (lower general secondary school, higher general secondary school, or secondary vocational school) and high (university-preparatory school, polytechnic, or university)

^b^Includes all Christian religions not belonging to the Catholic category

^c^Includes Coptic Christian and Greek- orthodox

^d^ Other languages used were Turkish (*n* = 20), English (*n* = 43), Arabic (*n* = 3) or Twi (*n* = 1). Written questionnaire was available in Dutch, English and Turkish

^e^Participants indicated to have received help to complete the questionnaire, i.e. partner (*n* = 48), daughter/son (*n* = 144) or different (*n* = 23)

^f^Girl is vaccinated for all vaccinations given between 0 and 4 years of age (without exceptions), which are DTaP: diphtheria-tetanus-acellular pertussis vaccine; HiB: Haemophilus influenzae type b vaccine; MMR: measles-mumps-rubella vaccine (NB; PCV: pneumococcal conjugate vaccine -- > was introduced in 2006 and therefore not applicable for this cohort.)

^**g**^Girl is vaccinated for all vaccinations given at 9 years of age (without exceptions), which are DTP: diphtheria-tetanus vaccine; MMR: measles-mumps-rubella vaccine

^h^Overall vaccination status between 0 and 9 years (HPV vaccination is offered at the age of 12–13 years)

Data are missing for gender (*n* = 52), age (*n* = 83), education (*n* = 76), religion (*n* = 80), received help (*n* = 57) and intention (*n* = 20). Data are missing for childhood and HPV vaccination status of those girls whose parents/guardians did not consent to obtain their vaccination status from Praeventis, the national database for monitoring the childhood vaccinations in the Netherlands)

*Abbreviations*: *IQR* interquartile range, *DPTP* diphtheria, pertussis, tetanus and poliomyelitis, *MMR* measles, mumps and rubella, *HPV* human papillomavirus, *SD* standard deviation. NL denotes participants with a Dutch ethnicity, SNA denotes participants with a Surinamese, Netherlands Antillean or Aruban ethnicity, MENA denotes participants with a Middle Eastern or North African ethnicity (including Turkish participants), Other denotes participants from all other ethnicities.

*p*-values/significance levels are indicated in the columns "p vs NL" and "p overall". The values thatare significant at a level *p* < 0.05 are now indicated in bold

Overall, the mean HPV vaccination intention was high (1.20, standard deviation, SD 1.17) but differed across ethnic groups (*p* < 0.001), with the lowest intention found in the MENA-group (mean 0.68; SD: 1.30). In the NL-group we found the largest fraction of daughters receiving two HPV vaccine doses (84%), while the SNA-group had the largest fraction of daughters that received only one dose (13%) and the MENA-group had the largest fraction of non-vaccinated daughters (41%). Overall past childhood vaccination uptake was 95% (Table 1).

### Determinants of HPV vaccination uptake by ethnic group

In the NL-group we found in multivariable analyses the following determinants to be significantly associated with HPV vaccination uptake (Table 2): intention (OR = 5.67; 95% CI = 4.10,7.85) and past childhood vaccination uptake (OR = 10.43; 95% CI = 3.05,35.69). In addition to the determinants found in the NL-group, we found habit strength (OR = 2.32; 95% CI = 1.13,4.78) to be significantly associated in the SNA-group. In the Other-group subjective norms (OR = 3.52; 95% CI = 1.28,9.67) and information processing (OR = 0.43; 95% CI = 0.22,0.83) were significantly associated. No additional determinants were found in the MENA-group. See Additional file 1: Table S3 for the selection of the variables used as input for the multivariable analyses.

**Table 2:** HPV vaccination uptake: multivariable logistic regression analyses of social-psychological, socio-demographic and other factors. HPV vaccination acceptability study among parents/guardians, in Amsterdam, the Netherlands, 2014

	Multivariable^a^							
	NL		SNA		MENA		Other	
	(*n* = 723)		(*n* = 126)		(*n* = 237)		(*n* = 223)	
	OR	95% CI	OR	95% CI	OR	95% CI	OR	95% CI
*Step 1*								
Intention	5.67	(4.10,7.85)	2.49	(1.30,4.76)	2.94	(2.10,4.11)	2.26	(1.37,3.71)
*Step 2*								
Subjective norms							3.52	(1.28,9.67)
*Step 3*								
Habit strength			2.32	(1.13,4.78)				
Information processing							0.43	(0.22,0.83)
Childhood vaccination ¥								
Not all	1							
All	10.43	(3.05,35.69)						
Multivariable model	pseudo-R^2^		pseudo-R^2^		pseudo-R^2^		pseudo-R^2^	
Step 1	0.54		0.20		0.23		0.22	
Step 1 + 2							0.25	
Step 1 + 2 + 3	0.56		0.28				0.29	

*Abbrevations*: *pseudo-R* ^*2*^ pseudo R squared, is a number that indicates how well the data fit a logistic regression model

NL denotes participants with a Dutch ethnicity, SNA denotes participants with a Surinamese, Netherlands Antillean or Aruban ethnicity, MENA denotes participants with a Middle Eastern or North African ethnicity (including Turkish participants), Other denotes participants from all other ethnicities

Interpretation of the OR of continuous determinants: If a determinant increases with one unit, we expect to see the odds to be HPV vaccinated (i.e. received 1 or 2 doses) to increase with the specified ratio for that determinant. For example, if among the Dutch intention increases with one unit, we expect to see the odds to be HPV vaccinated to increase 5.67 times

Interpretation of the OR of categorical determinants: the odds to be vaccinated are x-times higher in the non-reference categories compared to the reference category. For example, in the Dutch group if the daughter received all childhood vaccinations the odds to also become HPV vaccinated (i.e. received 1 or 2 doses) is 10.43 times higher compared to when the daughter did not receive all childhood vaccinations

^a^The multivariable analyses was executed by adding variables in three step: (step 1) intention, (step 2) proximal determinants, and (step 3) distal determinants. During each step backward selection was applied to reduce the number of determinants.

¥ Overall vaccination status for vaccinations taking place between 0 and 9 years old (HPV vaccination is offered at the age of 12-13 years)

The explained fraction (pseudo-R^2^) of HPV vaccination was 0.56 in the NL-group (0.54 by intention and an additional 0.02 by distal determinants); 0.28 in the SNA-group (0.20 by intention and an additional 0.08 by distal determinants); 0.23 in the MENA-group (all by intention); and 0.29 in the Other-group (0.22 by intention and an additional 0.03 by proximal determinants, and an additional 0.04 by distal determinants).

### Differences between determinants of HPV vaccination uptake by ethnic group

We tested whether the magnitude of the association of determinants of HPV vaccination uptake differed significantly in the non-Dutch groups compared to the NL-group (Table 3). The association of intention with uptake was significantly lower in the MENA-group (*p* = 0.020) and Other-group (*p* = 0.042) when compared to the NL-group; the association of subjective norms with uptake was significantly lower in the SNA-group (*p* = 0.025) and MENA-group (*p* = 0.002) when compared to the NL-group; habit strength was significantly lower in the MENA-group when compared to the NL-group (*p* < 0.001).

**Table 3:** Odds ratios for the association between key determinants and vaccination uptake among Dutch parents/guardians, and interaction between ethnic group and these determinants. HPV vaccination acceptability study among parents/guardians, in Amsterdam, the Netherlands, 2014

		Interaction effect						
		SNA vs Nl		MENA vs NL		Other vs NL		Overall
	NL-OR	xOR	*p*-value	xOR	*p*-value	xOR	*p*-value	*p*-value
Intention	5.19	0.54	*0.128*	0.58	***0.020***	0.57	***0.042***	*0.060*
Subjective norms	3.90	0.16	***0.025***	0.18	***0.002***	0.70	*0.604*	***0.006***
Habit strength	1.36	1.43	*0.415*	0.44	***<0.001***	0.58	***0.045***	***0.001***
Information processing	0.83	0.40	*0.141*	1.37	*0.265*	0.57	*0.153*	***0.043***
Childhood vaccination								
Not all	1							
All	8.52	0.33	*0.320*	0.65	*0.647*	0.30	*0.159*	*0.519*

The final multivariable model contains all variables significantly associated with HPV vaccination uptake in one of the ethnic groups

*p*-values < 0.05 are indicated in bold

NL denotes participants with a Dutch ethnicity, SNA denotes participants with a Surinamese, Netherlands Antillean or Aruban ethnicity, MENA denotes participants with a Middle Eastern or North African ethnicity (including Turkish participants), Other denotes participants from all other ethnicities

Interpretation of the OR of continuous determinants: If a determinant increases with one unit, we expect to see the odds to be HPV vaccinated to increase with the specified ratio for that determinant (i.e. received 1 or 2 doses). For example, if among the Dutch intention increases with one unit, we expect to see the odds to be HPV vaccinated to increase 5.19 times

Interpretation of the OR of categorical determinants: the odds to be vaccinated are x-times higher in the non-reference categories compared to the reference category. For example, in the Dutch group if the daughter received all childhood vaccinations the odds to also become HPV vaccinated (i.e. received 1 or 2 doses) is 8.52 times higher compared to when the daughter did not receive all childhood vaccinations. Interpretation of xOR: this is the odds ratio of the interaction factor. It indicates the factor with which the OR of the Dutch group should be multiplied to obtain the odds ratio for that determinants in that particular non-Dutch group. For example, the xOR for intention in the MENA group is 0.58. In order to obtain the odds ratio for the MENA-group for the effect of one step increase in the intention scale on vaccination uptake, one multiplies the OR of the Dutch group (5.19) with this xOR (0.58), resulting in an OR of 3.01. So the effect of intention on vaccination uptake is significantly (*p* = 0.020) less strong than in the Dutch group

### Relation between intention and uptake

Among the participants with a positive intention to vaccinate against HPV we observed that 4% in the NL-group, 11% in the SNA-Group (when compared to NL-group *p* = 0.002), 30% in the MENA-group (MENA- vs NL-group *p* < 0.001), and 11% in the Other-group (Other- vs NL-group *p* < 0.001) ultimately did not go for the HPV vaccination (inclined abstainers). Among the participants with a negative intention we observed that that 11% in the NL-group, 7% in the SNA-Group (SNA- vs NL-group *p* = 0.606), 6% in the MENA-group (MENA- vs NL-group *p* = 0.621) and 19% in the Other-group (Other- vs NL-group *p* = 0.298) did go for the HPV vaccination (disinclined actors).

### Determinants of HPV vaccination intention by ethnic group

In multivariable analyses we found the following determinants to be significantly associated with HPV vaccination intention in the NL-group (Table 4): (1) attitude (ß = 0.66; 95% CI = 0.57,0.75), (2) beliefs (ß = 0.36; 95% CI = 0.24,0.49), (3) subjective norms (ß = 0.49; 95% CI = 0.35,0.63), (4) ambivalence (ß = −0.08; 95% CI = −0.13,−0.03), (5) information processing (ß = −0.10; 95% CI = −0.15,−0.04), (6) subjective evaluation of the information (ß = 0.23; 95% CI = 0.12,0.34), (7) having an older daughter for whom they already had taken a decision about the HPV vaccination (the same decision was taken for the younger daughter) (ß = 0.92; 95% CI = 0.65,1.19), (8) education (intermediate education [ß = 0.14; 95% CI = 0.03,0.25] and higher education [ß = 0.13; 95% CI = 0.01,0.24] compared to lower education), and (9) religion (ß = −0.20; 95% CI = −0.30,−0.10). In addition to the determinants found in the NL-group, we found descriptive norms (ß = 0.13; 95% CI = 0.01,0.25) in the SNA-group, risk perception when deciding to not vaccinate (ß = 0.11; 95% CI = 0.01,0.21) and descriptive norms (ß = 0.12; 95% CI = 0.02,0.23) in the MENA-group, and descriptive norms (ß = 0.14; 95% CI = 0.05,0.23) and having had experience with (a prestage of) cervical cancer or knowing someone close with such an experience (ß = 0.20; 95% CI = 0.04,0.36) in the Other-group. See Additional file 1: Table S4 for the selection of the variables used as input for the multivariable analyses.

**Table 4:** HPV vaccination intention: multivariable linear regression analyses of socio-demographic, social-psychological and other factors. HPV vaccination acceptability study among parents/guardians, in Amsterdam, the Netherlands, 2014

	Multivariable^a^							
	NL		SNA		MENA		Other	
	(*n* = 723)		(*n* = 126)		(*n* = 237)		(*n* = 223)	
	ß	95% CI	ß	95% CI	ß	95% CI	ß	95% CI
*step 1*								
Attitude	0.66	(0.57,0.75)	0.70	(0.48,0.91)	1.03	(0.88,1.17)	0.71	(0.55,0.86)
Beliefs	0.36	(0.24,0.49)	0.28	(0.04,0.52)			0.27	(0.07,0.46)
Risk perception when not vaccinating					0.11	(0.01,0.21)		
Relative effectiveness	0.11	(0, 0.22)						
Subjective norms	0.49	(0.35,0.63)			0.27	(0.08,0.46)		
Descriptive norms			0.13	(0.01,0.25)	0.12	(0.02,0.23)	0.14	(0.05,0.23)
*step 2*								
Ambivalence towards the decision	−0.08	(−0.13,−0.03)						
Information processing	−0.10	(−0.15,−0.04)						
Evaluation of the HPV information	0.23	(0.12,0.34)	0.43	(0.21,0.66)			0.25	(0.06,0.44)
Past experience with vaccinating older daughter against HPV								
Older daughter not	REF						REF	
Older daughter partially/fully vaccinated	0.92	(0.65,1.19)					0.94	(0.33,1.54)
No older daughter	0.79	(0.53,1.05)					0.81	(0.21,1.40)
Past experience of someone close or him/herself with (prestage of) cervical cancer								
No							REF	
Yes							0.20	(0.04,0.36)
Education								
Low	REF							
Intermediate	0.14	(0.03,0.25)						
High	0.13	(0.01,0.24)						
Religion								
No religion	REF							
Religious	−0.20	(−0.30,−0.10)						
Multivariable model	R^2^		R^2^		R^2^		R^2^	
Step 1	0.73		0.61		0.75		0.63	
Step 1 + 2	0.77		0.66				0.66	

^a^The multivariable analyses was executed by adding variables in two step: (step 1) proximal determinants, and (step 2) distal determinants. During each step backward selection was applied to reduce the number of determinants. Please note, that when executing a stepwise multivariable analyses, variables significant (*p* < 0.05) in one step may not be significant when variables are added during subsequent steps

*Abbrevations*: *R* ^*2*^ R squared, is a number that indicates how well the data fit a linear regression model

NL denotes participants with a Dutch ethnicity, SNA denotes participants with a Surinamese, Netherlands Antillean or Aruban ethnicity, MENA denotes participants with a Middle Eastern or North African ethnicity (including Turkish participants), Other denotes participants from all other ethnicities

Interpretation of the coefficient of continuous determinants: if a determinant increases with one unit we expect to see an increase in intention with the coefficient specified for that determinant. For example, among the Dutch, if attitude increases with one unit, we expect intention to increase with 0.66

Interpretation of the coefficient of categorical determinants: the intention to be vaccinated is ß higher or lower in the non-reference category when compared to the reference category. For example, overall, Dutch participants that are highly educated have an intention that is 0.13 higher (on the scale of −2 to +2) when compared to those participants with a low education

The explained fraction of HPV intention (R^2^) was: 0.77 in NL-group (0.73 by proximal and an additional 0.04 by distal determinants), 0.66 in SNA-group (0.61 by proximal, an additional 0.05 by distal determinants), 0.75 in MENA-group (all by proximal determinants), and 0.66 in Other-group (0.63 by proximal and an additional 0.03 by distal determinants).

### Differences between determinants of HPV vaccination intention by ethnic group

We tested whether the magnitude of the association of determinants of HPV vaccination intention was significantly different in one of the non-Dutch groups when compared to the NL-group (Table 5). The association of subjective norms had a stronger impact on intention in the NL-group when compared to the SNA-group (*p* = 0.001) and Other-group (*p* = 0.001); when having taken the decision regarding the HPV vaccination for an older daughter, parents/guardians from the NL-group would more often take the same decision for the daughter now invited for vaccination, compared to parents from the SNA-group (p = 0.024) and MENA-group (*p* = 0.020). Religion plays a significantly stronger role on HPV vaccination intention in the NL-group when compared to the Other-group (*p* = 0.029).

**Table 5:** Regression coefficient for the association between key determinants and vaccination intention among Dutch parents/guardians, and interaction between ethnic group and these determinants. HPV vaccination acceptability study among parents/guardians, in Amsterdam, the Netherlands, 2014

		Interaction effect						
		SNA vs NL		MENA vs NL		Other vs NL		Overall
	NL-ß	∆ß	*p*-value	∆ß	*p*-value	∆ß	*p*-value	*p*-value
Attitude	0.77	−0.09	*0.307*	0.10	*0.070*	−0.12	*0.054*	***0.015***
Beliefs	0.36	−0.12	*0.268*	−0.12	*0.173*	−0.16	*0.051*	*0.182*
Risk perception when not vaccinating	0.06	−0.10	*0.113*	0.08	*0.119*	−0.08	*0.120*	***0.020***
Relative effectiveness	0.12	−0.17	*0.192*	−0.03	*0.735*	−0.19	***0.050***	*0.200*
Subjective norms	0.44	−0.40	***0.001***	−0.09	*0.335*	−0.34	***0.001***	***0.001***
Descriptive norms	0.09	−0.06	*0.383*	0.01	*0.806*	−0.05	*0.375*	*0.577*
Ambivalence towards the decision	−0.06	−0.01	*0.790*	0	*0.975*	0	*0.994*	*0.995*
Information processing	−0.11	0.02	*0.717*	0.09	*0.081*	0.06	*0.261*	*0.298*
Evaluation of the HPV information	0.24	0.08	*0.517*	−0.15	*0.112*	−0.09	*0.285*	*0.202*
Past experience with vaccinating older daughter against HPV								
Older daughter not	REF							***0.012***
Older daughter partially/fully vaccinated	0.95	−0.65	***0.024***	−0.54	***0.020***	−0.27	*0.431*	
No older daughter	0.81	−0.63	***0.017***	−0.69	***0.001***	−0.19	*0.561*	
Past experience of someone close or him/herself with (prestage of) cervical cancer								
No	REF							*0.093*
Yes	0.06	0.09	*0.509*	−0.21	*0.080*	0.11	*0.243*	
Education								
Low	REF							*0.282*
Intermediate	0.15	0.04	*0.760*	−0.02	*0.868*	−0.20	*0.090*	
High	0.15	0.16	*0.424*	−0.01	*0.942*	−0.19	*0.103*	
Religion								
No religion	REF							*0.187*
Religious	−0.20	0.08	*0.591*	0.09	*0.621*	0.22	***0.029***	

The final multivariable model contains all variables significantly associated with HPV vaccination intention in one of the ethnic groups

*p*-values <0.05 are indicated in bold

NL denotes participants with a Dutch ethnicity, SNA denotes participants with a Surinamese, Netherlands Antillean or Aruban ethnicity, MENA denotes participants with a Middle Eastern or North African ethnicity (including Turkish participants), Other denotes participants from all other ethnicities

Interpretation of the coefficient of continuous determinants: if a determinant increases with one unit we expect to see an increase in intention with the coefficient specified for that determinant. For example, among the Dutch, if attitude increases with one unit, we expect intention to increase with 0.77

Interpretation of the coefficient of categorical determinants: the intention to be vaccinated is ß higher or lower in the non-reference category when compared to the reference category. For example, overall, Dutch participants that are highly educated have an intention that is 0.15 higher (on the scale of −2 to +2) when compared to those participants with a low education

Interpretation of ∆ß: Delta Beta is the difference in the beta in one of the non-Dutch groups when compared to the NL-group. For example, for the variable Subjective Norms, the ∆ß in the SNA-group is −0.40, indicating that the slope is 0.40 lower in the SNA-group compared to the NL-group; this is a significant effect (*p* = 0.001)

### Complete case analyses

When comparing analyses restricted to the complete cases with analyses done on the imputed dataset we found similar results which did not affect our main conclusion (Additional file 1: Table S5 through S8).

## Discussion

In all ethnic groups we found intention to be the strongest predictor of HPV vaccination uptake, explaining the largest fraction of uptake. However, the total explained variance of uptake differed across ethnic groups. In the model with intention as outcome we found attitude to be significantly associated in all groups, attitude together with all proximal determinants explained most variance of intention. We did not find direct cues for targeting different determinants in the non-Dutch groups when designing interventions aiming to improve HPV vaccination intentions and uptake. As the strength of association of the determinants with both intention and uptake appeared similar across ethnic groups, except for a number of subtleties, mostly indicating stronger associations in the NL-group when compared to the non NL-groups.

In the NL-group intention explained a much larger fraction of the variability in HPV vaccination uptake (pseudo-R^2^ = 0.54) when compared to the other ethnic groups (pseudo-R^2^ of around 0.20). This discrepancy can be attributed to the higher proportion of participants with a positive intention who ultimately did not go for the vaccination (inclined abstainers [26]) in the SNA-group (11%) and MENA-group (30%) when compared to the NL-group (4%) -- a phenomenon that has been observed previously [27]. One might think that these discrepancies may have been caused by differential tendencies to respond in a socially desirable way to these questionnaires. However, we believe that this was not the case as we found a significantly lower intention in the MENA-group (please note this was not significantly lower in the SNA-group). An alternative explanation could be the presence of ‘logistical challenges’, yet the high coverage of other school-age vaccinations observed in the MENA-group and SNA-group (97% and 95%, respectively – see Table 1) suggest that this is not a plausible reason as these vaccinations are offered in the same way as the HPV vaccination and therefore require the same set of skills to ultimately get the vaccination. Another reason might be that parents/guardians from the MENA-group and SNA-group changed their mind in the time between completing the questionnaire and the appointment for receiving the HPV vaccination. We know that the HPV vaccination uptake in their social-circle is generally low and therefore they have a higher chance to encounter negative beliefs, norms, and role model behavior from their peers - it is possible that parents may therefore have a higher chance to change their mind.

We did not find large differences between the strength of association of the determinants on intention across ethnic groups, except for a couple of subtleties in which we found subjective norms and having an older daughter for which they already decided about the HPV vaccination to play a significantly stronger role in the NL-group. We found similar proportions of explained variance and similar determinants as in a previous study on native Dutch [10]: i.e. attitude, outcome beliefs, subjective norms, ambivalence, education, religion, and past (HPV) vaccination decision making. Additionally, we found the subjective evaluation of the information provided by the national HPV vaccination campaign to be significantly associated with intention in all groups except the MENA-group.

This is the first study to present longitudinal results regarding the association of intention to vaccinate against HPV and actual (rather than self-reported) HPV vaccination in an ethnically diverse sample. Since illiteracy and language barriers are known issues in our study population [28], we anticipated this by translating the questionnaire to other languages and by offering personal assistance with completing the questionnaire. The latter might have caused some bias, yet the direction of this bias remains to be elucidated. We executed in-depth qualitative research to develop a culturally sensitive questionnaire. We made every possible effort to recruit an ethnically diverse population and enable participants to complete the questionnaire (an action that might have been beyond their comfort range). Despite these efforts our response was 33%. Furthermore, girls whose parents/guardians participated in this study, had a higher HPV vaccination uptake than the average population. However, this difference in proportions is not likely to have biased our main results as they concern associations (determinants of HPV vaccination intention and uptake) instead of point estimates of uptake or intention.

We recommend future communication strategies to target social-psychological determinants of HPV vaccination intention and uptake. Furthermore, we may conclude that intervention programs should target the same determinants for all ethnic groups, although some determinants will have stronger effects in some groups than in others. Within the scope of these determinants, the process of HPV vaccination decision making has shown to be mainly attitudinally driven, however, we should not neglect processes of internalization of social normative/media influences. As HPV vaccination intention and uptake in the different ethnic groups is based on similar determinants, future interventions could employ similar behavior change methods (e.g. psychological inoculation or peer modeling). However, the selection and design of concrete applications or strategies to reach parents in the different ethnic populations may need tailoring. For example, a prerequisite for a successful communication is that non-Dutch speaking parents/guardians are able to understand and process the HPV vaccination information offered to them. In the MENA-group we found that 18% completed the questionnaire in another language than Dutch and that 43% completed the questionnaire with help of someone, we therefore advise to translate this information into their native language, and to use interaction-based approaches (personal communication or social media) so parents/guardians are able to ask questions and exchange their concerns about the HPV vaccination. This also provides opportunities to correct possible misperceptions before they become salient within a particular ethnic community. Further research is needed to understand the discrepancy between intention and behavior among the non-Dutch groups. Our research stresses the need of measuring actual uptake instead of intention in an ethnical diverse population.

## Conclusion

We confirmed that social-psychological factors are most decisive when parents/guardians make a decision regarding vaccinating their daughter against HPV. Based on the measured determinants we conclude that intervention programs can focus on the same determinants for all ethnic groups. Furthermore, we found the MENA-group and SNA-group to contain a large fraction of parents/guardians that had a positive intention to vaccinate yet ultimately decided not to go for the HPV vaccination of their daughter.

## Additional file

Additional file 1: Table S1. Overview of social-psychological scale measures used in the questionnaire for HPV vaccine acceptability among parents/guardians in Amsterdam, the Netherlands, 2014. **Table S2.** Response rate stratified by country of birth of the mother of the invited girl, HPV vaccination acceptability study in Amsterdam, the Netherlands, 2014. **Table S3.** HPV vaccination uptake: bivariable logistic regression analyses of social-psychological, socio-demographic and other factors. HPV vaccination acceptability study in Amsterdam, the Netherlands, 2014. **Table S4.** HPV vaccination intention: bivariable linear regression analyses of social-psychological, socio-demographic and other factors, HPV vaccination acceptability study in Amsterdam, the Netherlands, 2014. **Table S5.** HPV vaccination uptake: multivariable logistic regression analyses of complete cases of social-psychological, socio-demographic and other factors. HPV vaccination acceptability study among parents/guardians, in Amsterdam, the Netherlands, 2014. **Table S6.** Complete case analyses: Odds ratios for the association between key determinants and vaccination uptake among Dutch parents/guardians, and interaction between ethnic group and these determinants. HPV vaccination acceptability study among parents/guardians, in Amsterdam, the Netherlands, 2014. **Table S7.** HPV vaccination intention: multivariable linear regression analyses of complete cases of socio-demographic, social-psychological and other factors. HPV vaccination acceptability study among parents/guardians, in Amsterdam, the Netherlands, 2014. **Table S8.** Complete case analyses: Regression coefficient for the association between key determinants and vaccination intention among Dutch parents/guardians, and interaction between ethnic group and these determinants. HPV vaccination acceptability study among parents/guardians, in Amsterdam, the Netherlands, 2014. **Figure S1.** Schematic representation of key dates and periods: HPV vaccination acceptability study, Amsterdam 2014 The vertical line indicates the time. **Figure S2.** Flow diagram of the recruitment and response of parents/guardians and their daughters of the HPV vaccination acceptability study, Amsterdam 2014. **Figure S3.** Proportion of girls that received two HPV vaccine doses by neighborhood in the health district of the Public Health Service of Amsterdam. (DOCX 389 kb)

## Abbreviations

CI: Confidence interval
HPV: Human papillomavirus
IQR: Inter quartile range
MENA: Refers to the Middle Eastern and North African ethnical group
NIP: National Immunization Program
NL: Refers to the Dutch ethnical group
OR: Odds ratio
SD: Standard deviation
SNA: Refers to the Surinamese, Netherlands Antillean and Aruban ethnical group
