# Supplementary material for: A longitudinal study on determinants of HPV vaccination uptake in parents/guardians from different ethnic backgrounds in Amsterdam, the Netherlands
Source: BMC Public Health. 2017 Feb 21;17:220. doi: 10.1186/s12889-017-4091-4 (PMC5320738; doi:10.1186/s12889-017-4091-4)
Supplement: Additional file 1: Table S1. — Overview of social-psychological scale measures used in the questionnaire for HPV vaccine acceptability among parents/guardians in Amsterdam, the Netherlands, 2014. Table S2. Response rate stratified by country of birth of the mother of the invited girl, HPV vaccination acceptability study in Amsterdam, the Netherlands, 2014. Table S3. HPV vaccination uptake: bivariable logistic regression analyses of social-psychological, socio-demographic and other factors. HPV vaccination acceptability study in Amsterdam, the Netherlands, 2014. Table S4. HPV vaccination intention: bivariable linear regression analyses of social-psychological, socio-demographic and other factors, HPV vaccination acceptability study in Amsterdam, the Netherlands, 2014. Table S5. HPV vaccination uptake: multivariable logistic regression analyses of complete cases of social-psychological, socio-demographic and other factors. HPV vaccination acceptability study among parents/guardians, in Amsterdam, the Netherlands, 2014. Table S6. Complete case analyses: Odds ratios for the association between key determinants and vaccination uptake among Dutch parents/guardians, and interaction between ethnic group and these determinants. HPV vaccination acceptability study among parents/guardians, in Amsterdam, the Netherlands, 2014. Table S7. HPV vaccination intention: multivariable linear regression analyses of complete cases of socio-demographic, social-psychological and other factors. HPV vaccination acceptability study among parents/guardians, in Amsterdam, the Netherlands, 2014. Table S8. Complete case analyses: Regression coefficient for the association between key determinants and vaccination intention among Dutch parents/guardians, and interaction between ethnic group and these determinants. HPV vaccination acceptability study among parents/guardians, in Amsterdam, the Netherlands, 2014. Figure S1. Schematic representation of key dates and periods: HPV vaccination acceptability study, Amsterdam 2014 The ve [file 12889_2017_4091_MOESM1_ESM.docx]

Additional file 1 to:

**A longitudinal study on determinants of HPV vaccination uptake in parents/guardians from different ethnic backgrounds in Amsterdam, the Netherlands**

Catharina Johanna Alberts^1,2^, Maarten Schim van der Loeff^1,2^, Yvonne Hazeveld^3^, Hester de Melker^4^, Marcel van der Wal^5^, Astrid Nielen^3^, Fatima el Fakiri^5^, Maria Prins^1,2^, Theo Paulussen^6^

**1.** Public Health Service of Amsterdam (GGD), Department of Infectious Diseases, Department of Research, Amsterdam, the Netherlands
**2.** Department of Internal Medicine, Division of Infectious Diseases, Center for Infection and Immunity Amsterdam (CINIMA), Academic Medical Center (AMC), Amsterdam, the Netherlands

**3.** Public Health Service of Amsterdam (GGD), Department of Youth Health Service, Amsterdam, the Netherlands

**4.** National Institute of Public Health and the Environment (RIVM), Department of Epidemiology and Surveillance, Bilthoven, The Netherlands

**5.** Public Health Service of Amsterdam (GGD), Department of Epidemiology and Health Promotion, Amsterdam, the Netherlands

**6.** TNO (Netherlands Organization for Applied Scientific Research), Expertise Center Life Style, Leiden, the Netherlands

**Table of content Page**

Table S1 2

Table S2 10

Table S3 11

Table S4 13

Table S5 15

Table S6 16

Table S7 17

Table S8 19

Figure S1 21

Figure S2 23

Figure S3 24

**Table S1**. Overview of social-psychological scale measures used in the questionnaire for HPV vaccine acceptability among parents/guardians in Amsterdam, the Netherlands, 2014.

| **Measure** | **Item** | **Answer options** | **Scale** | **#item** | **Cron-bach’s α** |
| --- | --- | --- | --- | --- | --- |
| **HPV vaccination intention** | - Do you intend to have your daughter vaccinated against HPV?  - How likely is it that you will have your daughter vaccinated against HPV? | -2= Definitely not  -1= Probably not  0 = Maybe  1 = Probably yes  2 = Definitely yes | (-2 up to 2) | 2 | 0.96 |
| **Attitude** | - Getting my daughter vaccinated against HPV is… | -2= Very bad  -1= Bad  0 = Neither good nor bad  1 = Good  2 = Very Good | (-2 up to 2)  -2 = Very negative attitude  2 = Very positive attitude | 4 | 0.97 |
|  |  | -2= Very undesirable  -1= Undesirable  0 = Neither negative nor positive  1 = Desirable  2 = Very desirable |  |  |  |
|  |  | -2= Very negative  -1= Negative  0 = Neither negative nor positive  1 = Positive  2 = Very positive |  |  |  |
|  |  | -2= Very unimportant  -1= Unimportant  0 = Neither important nor unimportant  1 = Important  2 = Very important |  |  |  |
| **Beliefs about the HPV vaccination** | - According to my religion, it is better not to vaccinate my daughter against HPV.  - If the government vaccinates girls against HPV, then I assume that the HPV vaccination will be safe for my daughter.  - I only want to get my daughter vaccinated against HPV when she becomes sexually active.  - It is not proven that the HPV vaccination protects against cervical cancer.  - The long-term side effects of the HPV vaccination are unknown.  - The main reason that the HPV vaccination is offered, is because the pharmaceutical industry wants to make money. | -2 = Strongly agree  -1 = Agree  0 = Partly agree  1 = Disagree  2 = Strongly disagree  2nd item was coded as follows:  -2 = Strongly disagree  -1 = Disagree  0 = Partly agree  1 = Agree  2 =Strongly agree  *(For the analysis individuals with “Not applicable” were categorized as neutral)* | (-2 up to 2)  -2 = Negative beliefs regarding the HPV vaccination  2 = Positive beliefs regarding the HPV vaccination | 6 | 0.63 |
| **Negative outcome expectancies** | *If my daughter is vaccinated against HPV...*  - she will be very scared of being injected with a needle.  - she is very likely to suffer from unpleasant side effects shortly after being injected (e.g. headache, fever, pain at the injection site, etc.).  - she will suffer severe side effects later in life.  - she will very likely be unable to have children.  - her immune system will be damaged.  - she will practice unsafe sex in the future, as she will think she is protected against HPV anyway. | -2 = Strongly agree  -1 = Agree  0 = Partly agree  1 = Disagree  2 = Strongly disagree | (-2 up to 2)  -2 = Strong negative outcome expectancies  2 = Strong positive outcome expectancies | 6 | 0.70 |
| **Risk perception when not vaccinating** | Suppose you would not get your daughter vaccinated against HPV. In that case, how likely do you think it is that she will develop cervical cancer later on? | -2 = Very unlikely  -1 = Rather unlikely  0 = Neither likely nor unlikely  1 = Rather likely  2 = Very likely | N.A. | 1 | N.A. |
| **Anticipated regret about rejecting the HPV vaccination** | Suppose you would not get your daughter vaccinated against HPV and she develops cervical cancer later on. How much would you regret your decision not getting her vaccinated against HPV now? | -2 = No regret at all  -1 = Some regret  0 = Regret  1 = Much regret  2 = Very much regret | N.A. | 1 | N.A. |
| **Relative effectiveness of the HPV vaccination** | *How much will the methods listed below protect your daughter against cervical cancer?*  - HPV vaccination  - Always practice safe sex (by using a condom, for instance)  - Have sex with one single partner in her entire life  - Having a cervical smear taken every five years  - Always live a healthy life (e.g. eat healthy foods, plenty of exercise)  - Use alternative medicine (e.g. homeopathy) | 1 = Does not protect  2= Hardly protects  3 = Protects a little  4 = Protects much  5 = Protects very much | (-4 up to 4)  -4 = HPV vaccination is not effective at all when compared to the listed method  4 = HPV vaccination is much more effective when compared to the listed method | 6 | 0.80 |
| **Subjective norms towards the HPV vaccination** | *Normative beliefs*  Do the persons and organizations mentioned below think you should or should not vaccinate your daughter against HPV?  *Social referents:*  - Important leaders of my religion  - My partner  - My daughter  - My friends who also have a daughter who is invited to get  the HPV vaccination  - Good friends of my daughter  - My general practitioner  - Dutch governmental institutions  - Teachers at my daughter's school  - Parents originally from the same country as me, who also have a daughter who is invited to for the HPV vaccination | -2 = Definitely not  -1 = Probably not  0 = Probably not / Probably yes  1 = Probably yes  2 = Definitely yes  *(For the analysis individuals with “Not applicable” were categorized neutral).* | -2 = Negative  2 = Positive  Subjective norm was assessed by first multiplying normative beliefs and motivation to comply for each social reference, subsequently summing the products of the individual social references, then taking the average by dividing the number of social references measured, and finally rescaling it back to a range of -2 to 2. | 9 | 0.86 |
|  | *Motivation to comply*  To what extent do you take into account the opinion of the persons or organizations listed below when deciding to vaccinate or not vaccinate your daughter against HPV?  (Same social referents used) | 1 = Not at all  2 = Not much  3 = Neither much nor little  4 = Much  5 = Very Much  *(For the analysis individuals with “Not applicable” were categorized neutral)* |  |  |  |
| **Descriptive norms towards the HPV vaccination** | *How many of the persons mentioned*  *below do you believe will vaccinate their daughter against*  *HPV?*  - My friends who also have a daughter who is invited to for the HPV vaccination  - Parents of good friends of my daughter who also have received the invitation for the HPV vaccination  - Parents originally from the same country as me, who also have a daughter who is invited for the HPV vaccination | 1 =None  2 = Less than half  3 = About half  4 = More than half  5 = All | (1 up to 5)  1 = None  5 = All | 3 | 0.94 |
| **Self efficacy convincing others when they have another opinion about the HPV vaccination** | *Suppose you already decided whether to vaccinate or not vaccinate your daughter against HPV. Would you be able to convince others of your opinion in the situations described below?*  - I will be able to convince my daughter about my choice if my daughter does not agree with me  - I will be able to convince my partner about my choice if my partner does not agree with me, but my daughter does.  - I will be able to convince my daughter about my choice if my daughter does not agree with me, but my partner does.  - I will be able to convince my partner and my daughter about my choice if both do not agree with me. | -2 = I definitely will not be  able to  -1 = I probably  will not be able to  0 = I may be able to/ May not  be able to  1 = I probably will be  able to  2 = I definitely will be  able to | (-2 up to 2)  -2 = Definitely not be able  2 = Definitely be able | No prt:1  Prt:4 | No Prt = N.A.  Prt =  0.86 |
| **Self efficacy expectation when dealing with information processing regarding the HPV vaccination** | *I will be able to…*  - read by myself the information on the HPV vaccine I received last week.  - understand the information on the HPV vaccination I  received last week.  - find other sources that provide additional information on the advantages and disadvantages of the HPV vaccination (for instance, on the internet or from the general practitioner). | -2 = I definitely will not be  able to  -1 = I probably  will not be able to  0 = I may be able to/ May not  be able to  1 = I probably will be  able to  2 = I definitely will be  able to | (-2 up to 2)  -2 = I definitely will not be able to  2 = I definitely will be  able to | 3 | 0.91 |
| **Knowledge about the HPV vaccination** | - The HPV vaccination is mandatory (incorrect)  - The HPV vaccination is injected into the uterus (incorrect)  - The HPV vaccination provides full protection against cervical cancer (incorrect)  - The HPV vaccination contains substances that may cause cancer (incorrect)  - HPV is sexually transmitted (correct)  - If you have HPV you will always notice this (incorrect)  - The Dutch government advises women vaccinated against HPV to still participate in the cervical cancer screening programme (correct) | -1 = incorrect  0 = don’t know  1 = correct | (-7 up to 7)  -7 = incorrect  7 = correct | 7 | N.A. |
| **Confidence in authorities involved in the HPV vaccination** | *If the persons or organization mentioned below would provide you with advice on whether to vaccinate or not vaccinate your daughter against HPV, how much faith would you have in their advice?*  - The general practitioner  - Teachers at school  - Dutch governmental institutions (e.g. the GGD and the RIVM)  - Science (e.g. universities) | -2 = Very little faith  -1 = Little faith  0 = Neither a lot nor little faith  1 = Some faith  2 = A lot of faith | (-2 up to 2)  -2 = Very little faith  2 = A lot of faith | 4 | 0.71 |
| **Ambivalence towards the HPV vaccination decision** | *When deciding whether to get my daughter vaccinated against HPV…*  - I am torn between the advantages and disadvantages of doing so.  - I experience both positive and negative feelings. | -2 = Strongly disagree  -1 = Disagree  0 = Partly agree  1 = Agree  2 = Strongly | (-2 up to 2)  -2 = Strongly disagree  2 = Strongly agree | 2 | 0.89 |
| **Habit strength** | *Vaccinating my daughter against HPV…*  - is not a matter that requires much thought.  - goes without saying. | -2 = Strongly disagree  -1 = Disagree  0 = Partly agree  1 = Agree  2 = Strongly | (-2 up to 2)  -2 = Strongly disagree  2 = Strongly agree | 2 | 0.85 |
| **Information processing** | *Before I decide whether to get my daughter vaccinated or not vaccinated against HPV…*  - I will first think long and hard about it.  - I will first search for more information on the HPV vaccination. | -2 = Strongly disagree  -1 = Disagree  0 = Partly agree  1 = Agree  2 = Strongly agree | (-2 up to 2)  -2 = Strongly disagree  2 = Strongly agree | 2 | 0.82 |
| **Amount of information processed** | - Have you read the information regarding the HPV  vaccination you received last week? | 0 = I have not received the information  1 = I have not (yet) read the information  2 = I have read some of the information  3 = I have read most of the information  4 = I have read all of the information | 0 = Not read anything  4 = Read all the information | 1 |  |
|  | - Have you visited the RIVM website  ([www.rivm.nl/rijksvaccinatieprogramma](http://www.rivm.nl/rijksvaccinatieprogramma))?  *Information processing of the leaflet and RIVM website were not combined because Cronbach's alpha was <0.60 (namely 0.41).* | 0 = I do not know the website  1 = I have not (yet) visited the  website  2 = I have visited some parts  3= I have visited most parts  4 = I have visited the entire website | 0 = Not read anything  4 = Read all the information | 1 |  |
| **Evaluation of the HPV information** | What do you think about the information regarding the  HPV vaccination you received last week?  What do you think about the information regarding the  HPV vaccination on the RIVM website? | -2= Far too little/much focus on the **advantages**  0 = Too little/much focus on the **advantages**  2 = Enough focus on the  **advantages** | -2 = Extremely negative about how the information is presented  2 = Extremely positive about how the information is presented | 5 | 0.82 |
|  |  | -2 = Far too little/much focus on the **disadvantages**  0 = Too little/much focus on the **disadvantages**  2 = Enough focus on the  **disadvantages** |  |  |  |
|  |  | -2 = Far too little/much focus on the **amount** of information  0 = Too little/much focus on the **amount** of information  2 = Enough focus on the  **amount** of information |  |  |  |
|  |  | -2 = Not reliable at all  -1 = Not reliable  0 = A bit reliable  1 = Reliable  2 = Very reliable |  |  |  |
|  |  | -2 = Not clear at all  -1 = Not clear  0 = A bit clear  1 = Clear  2 = Very clear |  |  |  |
| **Past experience with vaccinating older daughter against HPV** | Do you have an older daughter (or daughters), born  between 1993-2000, who was invited to be vaccinated  against HPV in previous years?  Was this older daughter (daughters) vaccinated against  HPV? | 1 = Older daughter not vaccinated  2 = Older daughter partially/fully vaccinated  3 = No older daughter | N.A. | 1 | N.A. |
| **Past experience of someone close or him/herself with (prestage of) cervical cancer** | Have you ever had (a preliminary stage of) cervical  cancer?  Has someone in your direct environment had  (a preliminary stage of) cervical cancer? | 1 = No  2 = Yes  If answered yes to one of the two questions a person was categorized as yes. | N.A. | 1 | N.A. |
| **Location HPV vaccination** | To what location would you prefer to go to when vaccinating your daughter against HPV?  *You may tick multiple locations.* | 1 = A sports centre (as is currently the case)  2 = My daughter's school  3= The child health clinic of the GGD  4 = General practitioner  5 = Nowhere, I do not want my daughter to get vaccinated against HPV  6= Other, i.e. …. | N.A. |  |  |

| **Table S2**. Response rate stratified by country of birth of the mother of the invited girl, HPV vaccination acceptability study in Amsterdam, the Netherlands, 2014. | | | | | |
| --- | --- | --- | --- | --- | --- |
|  | **Participants** | | **Non-participants** | | **Total** |
|  | n | % | n | % | n |
| NL | 820 | 37% | 1,403 | 63% | 2,223 |
| SNA | 113 | 31% | 254 | 69% | 367 |
| MENA | 204 | 20% | 809 | 80% | 1,013 |
| Other | 192 | 34% | 377 | 66% | 569 |
| **Total population** | **1,329** | **32%** | **2,843** | **68%** | **4,172** |
| NL denotes mothers with the Netherlands as country of birth, SNA denotes mothers with Surinam, Netherlands Antilles, or Aruba as country of birth, MENA denotes a country of birth of the mother of one of the Middle Eastern or North African countries (including Turkey), Other denotes participants from all other countries. | | | | | |
| Please note that the four groups depicted in the table are based on country of birth of the mother of the invited girl, rather than the categorization based on ethnicity used throughout the manuscript. | | | | | |
| The total number of persons indicated as participants is not identical to the 1,362 persons indicated as participants in Figure S1. The reason for this is that of the 1,362 participants the country of birth of the mother was missing for 18 mothers and the studynumber was missing for 15 mothers (and could therefore not be identified in the database) (i.e. 1,362-18-15=1,329) | | | | | |
| Country of birth of the mother was missing for 26 non-participants. Considering the missing information on country of birth of the mother, this results in a total of 4.172 persons eligible for comparison (i.e. 4,216-18-26=4,172). Please note that the 15 parents which could not be identified because his/her study number was missing are now included in the non-participant group. | | | | | |

| **Table S3**. HPV vaccination uptake: bivariable logistic regression analyses of social-psychological, socio-demographic and other factors. HPV vaccination acceptability study in Amsterdam, the Netherlands, 2014. | | | | | | | | | | | | | | | | |
| --- | --- | --- | --- | --- | --- | --- | --- | --- | --- | --- | --- | --- | --- | --- | --- | --- |
|  | NL | | |  | SNA | | |  | MENA | | |  | Other | | | |
|  | (n=723) | | |  | (n=126) | | |  | (n=237) | | |  | (n=223) | | | |
|  | Bivariable | | |  | Bivariable | | |  | Bivariable | | |  | Bivariable | | |  |
|  | OR | 95% CI | pseudo-R^2^ | | OR | 95% CI | pseudo-R^2^ | | OR | 95% CI | pseudo-R^2^ | | OR | 95% CI | pseudo-R^2^ | |
| ***Intention*** | 5.62 | (4.11,7.68) | 0.536 |  | 3.30 | (1.69,6.44) | 0.202 |  | 2.94 | (2.10,4.11) | 0.226 |  | 3.29 | (2.10,5.14) | 0.218 |  |
| ***Proximal determinants*** |  |  |  |  |  |  |  |  |  |  |  |  |  |  |  |  |
| **Attitude** | 15.59 | (9.23,26.33) | 0.459 |  | 5.65 | (2.07,15.47) | 0.188 |  | 3.25 | (2.09,5.07) | 0.136 |  | 4.28 | (2.39,7.66) | 0.165 |  |
| **Beliefs** | 20.74 | (11.70,36.76) | 0.339 |  | 5.30 | (1.82,15.43) | 0.122 |  | 2.10 | (1.24,3.55) | 0.031 |  | 4.23 | (2.05,8.75) | 0.097 |  |
| **Negative outcome expectations** | 3.84 | (2.47,5.95) | 0.073 |  | 2.22 | (0.99,4.99) | 0.039 |  | 1.43 | (0.88,2.30) | 0.010 |  | 2.08 | (1.04,4.13) | 0.024 |  |
| **Risk perception no HPV vac.** | 2.37 | (1.83,3.08) | 0.076 |  | 1.70 | (0.97,2.97) | 0.042 |  | 1.58 | (1.14,2.17) | 0.034 |  | 1.32 | (0.87,2.00) | 0.009 |  |
| **Anticipated regret rejecting** | 2.61 | (2.13,3.21) | 0.196 |  | 1.89 | (1.17,3.05) | 0.086 |  | 1.52 | (1.21,1.92) | 0.046 |  | 1.73 | (1.26,2.39) | 0.062 |  |
| **Relative effectiveness** | 9.79 | (5.62,17.05) | 0.144 |  | 2.66 | (0.81,8.71) | 0.030 |  | 2.13 | (1.23,3.68) | 0.030 |  | 2.64 | (1.22,5.69) | 0.033 |  |
| **Subjective norms** | 62.23 | (29.30,132.17) | 0.315 |  | 1.66 | (0.66,4.15) | 0.011 |  | 2.67 | (1.56,4.56) | 0.056 |  | 5.29 | (2.41,11.64) | 0.106 |  |
| **Descriptive norms** | 3.09 | (2.25,4.23) | 0.095 |  | 1.30 | (0.79,2.16) | 0.010 |  | 1.84 | (1.38,2.45) | 0.077 |  | 1.61 | (1.16,2.24) | 0.040 |  |
| **Self-efficacy convincing** | 2.10 | (1.53,2.87) | 0.041 |  | 1.63 | (0.86,3.11) | 0.028 |  | 1.11 | (0.81,1.52) | 0.002 |  | 1.28 | (0.82,1.99) | 0.006 |  |
| **Self-efficacy** | 0.82 | (0.51,1.31) | 0.002 |  | 0.97 | (0.60,1.57) | 0.001 |  | 1.06 | (0.85,1.32) | 0.001 |  | 0.82 | (0.58,1.16) | 0.007 |  |
| ***Distal determinants*** |  |  |  |  |  |  |  |  |  |  |  |  |  |  |  |  |
| **Knowledge** | 1.14 | (0.98,1.33) | 0.005 |  | 0.97 | (0.76,1.23) | 0.003 |  | 0.97 | (0.85,1.10) | 0.001 |  | 0.99 | (0.85,1.15) | 0.001 |  |
| **Confidence in authorities** | 10.38 | (6.16,17.48) | 0.218 |  | 1.83 | (0.79,4.20) | 0.025 |  | 1.85 | (1.13,3.02) | 0.032 |  | 1.88 | (1.01,3.48) | 0.022 |  |
| **Ambivalence towards the decision** | 1.77 | (1.45,2.17) | 0.056 |  | 1.49 | (0.92,2.41) | 0.030 |  | 1.07 | (0.82,1.38) | 0.001 |  | 1.62 | (1.12,2.33) | 0.039 |  |
| **Habit strength** | 3.34 | (2.61,4.28) | 0.238 |  | 2.85 | (1.53,5.33) | 0.176 |  | 1.13 | (0.88,1.46) | 0.003 |  | 2.03 | (1.38,2.98) | 0.078 |  |
| **Information processing** | 0.33 | (0.23,0.47) | 0.091 |  | 0.36 | (0.15,0.87) | 0.084 |  | 0.90 | (0.68,1.20) | 0.002 |  | 0.36 | (0.21,0.63) | 0.095 |  |
| **Information offered by the government** |  |  |  |  |  |  |  |  |  |  |  |  |  |  |  |  |
| Amount processed from leaflet | 0.96 | (0.79,1.15) | 0 |  | 0.92 | (0.55,1.52) | 0.003 |  | 1.16 | (0.91,1.48) | 0.005 |  | 0.76 | (0.55,1.06) | 0.014 |  |
| Amount processed from website | 0.75 | (0.62,0.91) | 0.015 |  | 1.07 | (0.68,1.68) | 0.002 |  | 1.26 | (0.97,1.64) | 0.013 |  | 0.92 | (0.64,1.33) | 0.001 |  |
| Evaluation of the HPV information | 5.11 | (3.46,7.55) | 0.191 |  | 3.10 | (1.16,8.27) | 0.062 |  | 1.69 | (1.02,2.81) | 0.022 |  | 2.18 | (1.15,4.16) | 0.046 |  |
| **Past experience with vaccinating older daughter against HPV** | | |  |  |  |  |  |  |  |  |  |  |  |  |  |  |
| Older daughter not vaccinated |  |  | 0.124 |  |  |  | 0.013 |  |  |  | 0.066 |  |  |  | 0.033 |  |
| Older daughter partially/fully vaccinated | 131.3 | (29.65,581.29) | |  | 2.01 | (0.26,15.20) |  |  | 18.02 | (3.26,99.62) |  |  | 18.41 | (1.58,215.10) |  |  |
| No older daughter | 35.05 | (10.29,119.46) | |  | 2.59 | (0.46,14.61) |  |  | 3.30 | (1.24,8.78) |  |  | 12.43 | (1.23,125.36) |  |  |
| **Past experience of someone close or him/herself with (prestage of) cervical cancer** | | | | | |  |  |  |  |  |  |  |  |  |  |  |
| No | 1 |  | 0.002 |  | 1 |  | 0.002 |  | 1 |  | 0.002 |  | 1 |  | 0.001 |  |
| Yes | 1.29 | (0.85,1.96) |  |  | 1.13 | (0.34,3.81) |  |  | 1.32 | (0.67,2.59) |  |  | 1.06 | (0.50,2.23) |  |  |
| **Childhood vaccination ¥** |  |  |  |  |  |  |  |  |  |  |  |  |  |  |  |  |
| Not all | 1 |  | 0.054 |  | 1 |  | 0.068 |  | 1 |  | 0.001 |  | 1 |  | 0.038 |  |
| All | 9.81 | (4.24,22.69) |  |  | 7.12 | (1.62,31.31) |  |  | 1.32 | (0.34,5.17) |  |  | 3.86 | (1.41,10.56) |  |  |
| **Socio-demographic characteristics** |  |  |  |  |  |  |  |  |  |  |  |  |  |  |  |  |
| **Gender** |  |  |  |  |  |  |  |  |  |  |  |  |  |  |  |  |
| Female | 1 |  | 0 |  | 1 |  | 0.001 |  | 1 |  | 0.001 |  | 1 |  | 0.008 |  |
| Male | 0.86 | (0.44,1.70) |  |  | 0.94 | (0.18,4.98) |  |  | 0.83 | (0.40,1.72) |  |  | 2.42 | (0.53,11.06) |  |  |
| **Age (years)** |  |  |  |  |  |  |  |  |  |  |  |  |  |  |  |  |
| ≤ 43 year | 1 |  | 0.009 |  | 1 |  | 0.003 |  | 1 |  | 0.009 |  | 1 |  | 0.021 |  |
| 44 , 47 year | 1.54 | (0.93,2.55) |  |  | 1.00 | (0.29,3.47) |  |  | 1.72 | (0.73,4.05) |  |  | 1.44 | (0.64,3.22) |  |  |
| ≥ 48 year | 1.80 | (1.04,3.11) |  |  | 0.92 | (0.26,3.23) |  |  | 0.97 | (0.47,1.99) |  |  | 2.54 | (0.97,6.61) |  |  |
| **Education** |  |  |  |  |  |  |  |  |  |  |  |  |  |  |  |  |
| Low | 1 |  | 0.018 |  | 1 |  | 0.019 |  | 1 |  | 0.024 |  | 1 |  | 0.002 |  |
| Intermediate | 0.87 | (0.53,1.42) |  |  | 0.57 | (0.18,1.85) |  |  | 1.99 | (0.96,4.12) |  |  | 1.13 | (0.48,2.68) |  |  |
| High | 2.02 | (1.13,3.61) |  |  | 0.50 | (0.08,3.15) |  |  | 2.43 | (1.01,5.82) |  |  | 1.28 | (0.53,3.13) |  |  |
| **Religion** |  |  |  |  |  |  |  |  |  |  |  |  |  |  |  |  |
| No religion | 1 |  | 0.012 |  | 1 |  | 0.005 |  | 1 |  | 0.021 |  | 1 |  | 0.001 |  |
| Religious | 0.54 | (0.35,0.84) |  |  | 1.60 | (0.47,5.51) |  |  | 0.17 | (0.03,1.12) |  |  | 1.19 | (0.59,2.43) |  |  |
| pseudo-R^2^ = pseudo R squared, is a number that indicates how well the data fit a logistic regression model. | | | | | | | |  |  |  |  |  |  |  |  |  |
| NL denotes participants with a Dutch ethnicity, SNA denotes participants with a Surinamese, Netherlands Antillean or Aruban ethnicity, MENA denotes participants with a Middle Eastern or North African ethnicity (including Turkish participants), Other denotes participants from all other ethnicities. | | | | | | | | | | | | | | | | |

| **Table S4**. HPV vaccination intention: bivariable linear regression analyses of social-psychological, socio-demographic and other factors, HPV vaccination acceptability study in Amsterdam, the Netherlands, 2014. | | | | | | | | | | | | | | | | |
| --- | --- | --- | --- | --- | --- | --- | --- | --- | --- | --- | --- | --- | --- | --- | --- | --- |
|  | NL | | | | SNA | | |  | MENA | | |  | Other | | | |
|  | (n=723) | | | | (n=126) | | |  | (n=237) | | |  | (n=223) | | | |
|  | Bivariable | | |  | Bivariable | | |  | Bivariable | | |  | Bivariable | | |  |
|  | ß | 95% CI | R^2^ |  | ß | 95% CI | R^2^ |  | ß | 95% CI | R^2^ |  | ß | 95% CI | R^2^ |  |
| ***Proximal determinants*** |  |  |  |  |  |  |  |  |  |  |  |  |  |  |  |  |
| **Attitude** | 1.18 | (1.12,1.24) | 0.671 |  | 1.03 | (0.87,1.19) | 0.575 |  | 1.28 | (1.17,1.38) | 0.715 |  | 1.01 | (0.90,1.13) | 0.596 |  |
| **Beliefs** | 1.46 | (1.35,1.57) | 0.478 |  | 0.94 | (0.68,1.20) | 0.307 |  | 1.26 | (1.00,1.51) | 0.309 |  | 1.06 | (0.87,1.25) | 0.372 |  |
| **Negative outcome expectations** | 0.90 | (0.75,1.06) | 0.154 |  | 0.26 | (-0.01,0.53) | 0.029 |  | 0.56 | (0.30,0.81) | 0.082 |  | 0.43 | (0.19,0.66) | 0.055 |  |
| **Risk perception no HPV vac.** | 0.44 | (0.34,0.54) | 0.096 |  | 0.26 | (0.10,0.42) | 0.082 |  | 0.57 | (0.41,0.72) | 0.185 |  | 0.22 | (0.08,0.36) | 0.040 |  |
| **Anticipated regret rejecting** | 0.53 | (0.47,0.59) | 0.294 |  | 0.38 | (0.23,0.52) | 0.189 |  | 0.59 | (0.47,0.71) | 0.317 |  | 0.38 | (0.28,0.49) | 0.191 |  |
| **Relative effectiveness** | 1.17 | (1.00,1.33) | 0.206 |  | 0.58 | (0.22,0.92) | 0.086 |  | 0.98 | (0.69,1.26) | 0.178 |  | 0.68 | (0.43,0.94) | 0.115 |  |
| **Subjective norms** | 1.88 | (1.71,2.05) | 0.413 |  | 0.50 | (0.20,0.80) | 0.083 |  | 1.24 | (1.00,1.47) | 0.332 |  | 0.84 | (0.60,1.08) | 0.183 |  |
| **Descriptive norms** | 0.77 | (0.65,0.89) | 0.189 |  | 0.37 | (0.20,0.54) | 0.134 |  | 0.71 | (0.59,0.83) | 0.389 |  | 0.35 | (0.22,0.47) | 0.131 |  |
| **Self-efficacy convincing** | 0.43 | (0.31,0.56) | 0.064 |  | 0.31 | (0.11,0.52) | 0.074 |  | 0.40 | (0.22,0.56) | 0.086 |  | 0.15 | (-0.01,0.32) | 0.016 |  |
| **Self-efficacy** | 0.04 | (-0.11,0.19) | 0 |  | 0.08 | (-0.07,0.23) | 0.010 |  | 0.08 | (-0.05,0.21) | 0.006 |  | 0.02 | (-0.09,0.13) | 0.001 |  |
| ***Distal determinants*** |  |  |  |  |  |  |  |  |  |  |  |  |  |  |  |  |
| **Knowledge** | 0.09 | (0.03,0.16) | 0.010 |  | 0 | (-0.07,0.07) | 0 |  | -0.04 | (-0.11,0.03) | 0.004 |  | -0.03 | (-0.08,0.03) | 0.004 |  |
| **Confidence in authorities** | 1.12 | (0.99,1.25) | 0.290 |  | 0.39 | (0.14,0.63) | 0.075 |  | 0.86 | (0.63,1.08) | 0.219 |  | 0.54 | (0.34,0.75) | 0.113 |  |
| **Ambivalence towards the decision** | 0.37 | (0.29,0.45) | 0.108 |  | 0.15 | (0,0.29) | 0.032 |  | 0.33 | (0.18,0.48) | 0.079 |  | 0.29 | (0.17,0.41) | 0.096 |  |
| **Habit strength** | 0.59 | (0.53,0.65) | 0.346 |  | 0.36 | (0.22,0.50) | 0.182 |  | 0.45 | (0.30,0.59) | 0.140 |  | 0.52 | (0.42,0.62) | 0.311 |  |
| **Information processing** | -0.46 | (-0.56,-0.37) | 0.121 |  | -0.17 | (-0.36,0.01) | 0.028 |  | -0.19 | (-0.36,-0.01) | 0.020 |  | -0.27 | (-0.40,-0.14) | 0.069 |  |
| **Information offered by the government** |  |  |  |  |  |  |  |  |  |  |  |  |  |  |  |  |
| Amount processed from leaflet | -0.05 | (-0.12,0.02) | 0.002 |  | 0.18 | (0.02,0.33) | 0.046 |  | -0.02 | (-0.17,0.13) | 0 |  | -0.07 | (-0.18,0.04) | 0.007 |  |
| Amount processed from website | -0.17 | (-0.25,-0.09) | 0.025 |  | -0.05 | (-0.19,0.09) | 0.004 |  | 0.15 | (0,0.29) | 0.017 |  | -0.16 | (-0.29,-0.03) | 0.028 |  |
| Evaluation of the HPV information | 1.13 | (0.99,1.26) | 0.376 |  | 0.91 | (0.61,1.22) | 0.276 |  | 0.55 | (0.26,0.85) | 0.087 |  | 0.69 | (0.41,0.96) | 0.200 |  |
| **Past experience with vaccinating older daughter against HPV** | | |  |  |  |  |  |  |  |  |  |  |  |  |  |  |
| Older daughter not | REF |  | 0.181 |  | REF |  | 0.084 |  | REF |  | 0.090 |  | REF |  | 0.066 |  |
| Older daughter partially/fully vaccinated | 2.94 | (2.48,3.41) |  |  | 1.22 | (0.47,1.97) |  |  | 1.60 | (0.90,2.31) |  |  | 1.77 | (0.80,2.74) |  |  |
| No older daughter | 2.55 | (2.11,2.99) |  |  | 0.77 | (0.11,1.43) |  |  | 0.98 | (0.43,1.53) |  |  | 1.42 | (0.48,2.36) |  |  |
| **Past experience of someone close or him/herself with (prestage of) cervical cancer** | | | |  |  |  |  |  |  |  |  |  |  |  |  |  |
| No | REF |  | 0.007 |  | REF |  | 0.002 |  | REF |  | 0.005 |  | REF |  | 0.009 |  |
| Yes | 0.20 | (0.02,0.38) |  |  | 0.10 | (-0.28,0.48) |  |  | -0.22 | (-0.64,0.20) |  |  | 0.18 | (-0.08,0.44) |  |  |
| **Childhood vaccination** |  |  |  |  |  |  |  |  |  |  |  |  |  |  |  |  |
| Not all | REF |  | 0.037 |  | REF |  | 0.100 |  | REF |  | 0.025 |  | REF |  | 0.041 |  |
| All | 1.13 | (0.59,1.66) |  |  | 1.06 | (0.32,1.79) |  |  | -1.03 | (-2.06,0) |  |  | 0.62 | (0.08,1.16) |  |  |
| **Socio-demographic characteristics** |  |  |  |  |  |  |  |  |  |  |  |  |  |  |  |  |
| **Gender** |  |  |  |  |  |  |  |  |  |  |  |  |  |  |  |  |
| Female | REF |  | 0 |  | REF |  | 0.001 |  | REF |  | 0 |  | REF |  | 0.013 |  |
| Male | 0.07 | (-0.22,0.35) |  |  | 0.08 | (-0.46,0.61) |  |  | 0.03 | (-0.42,0.48) |  |  | 0.36 | (-0.06,0.77) |  |  |
| **Age (years)** |  |  |  |  |  |  |  |  |  |  |  |  |  |  |  |  |
| ≤ 43 year | REF |  | 0.008 |  | REF |  | 0.013 |  | REF |  | 0.002 |  | REF |  | 0.011 |  |
| 44 , 47 year | 0.18 | (-0.03,0.40) |  |  | 0.15 | (-0.25,0.54) |  |  | 0.12 | (-0.37,0.61) |  |  | 0.18 | (-0.13,0.48) |  |  |
| ≥ 48 year | 0.25 | (0.03,0.47) |  |  | -0.14 | (-0.57,0.28) |  |  | 0.07 | (-0.38,0.53) |  |  | -0.08 | (-0.40,0.24) |  |  |
| **Education** |  |  |  |  |  |  |  |  |  |  |  |  |  |  | 0.001 |  |
| Low | REF |  | 0.020 |  | REF |  | 0.008 |  | REF |  | 0.012 |  | REF |  |  |  |
| Intermediate | -0.11 | (-0.33,0.10) |  |  | -0.17 | (-0.57,0.22) |  |  | 0.17 | (-0.28,0.62) |  |  | -0.04 | (-0.36,0.27) |  |  |
| High | 0.27 | (0.05,0.48) |  |  | 0 | (-0.57,0.58) |  |  | 0.39 | (-0.10,0.89) |  |  | -0.06 | (-0.37,0.25) |  |  |
| **Religion** |  |  |  |  |  |  |  |  |  |  |  |  |  |  |  |  |
| No religion | REF |  | 0.018 |  | REF |  | 0.022 |  | REF |  | 0.011 |  | REF |  | 0 |  |
| Religious | -0.36 | (-0.55,-0.16) |  |  | 0.38 | (-0.07,0.83) |  |  | -0.59 | (-1.32,0.14) |  |  | -0.03 | (-0.29,0.23) |  |  |
| R^2^ = R squared, is a number that indicates how well the data fit a linear regression model. | | | | | |  |  |  |  |  |  |  |  |  |  |  |
| NL denotes participants with a Dutch ethnicity, SNA denotes participants with a Surinamese, Netherlands Antillean or Aruban ethnicity, MENA denotes participants with a Middle Eastern or North African ethnicity (including Turkish participants), Other denotes participants from all other ethnicities. | | | | | | | | | | | | | | | |  |

| **Table S5**.HPV vaccination uptake: multivariable logistic regression analyses of complete cases of social-psychological, socio-demographic and other factors. HPV vaccination acceptability study among parents/guardians, in Amsterdam, the Netherlands, 2014. | | | | | | | | | | | | |
| --- | --- | --- | --- | --- | --- | --- | --- | --- | --- | --- | --- | --- |
|  |  | **Multivariable*** | | | | | | | | | | |
|  |  | **NL** | |  | **SNA** | |  | **MENA** | |  | **Other** | |
|  |  | (n=644) | |  | (n=100) | |  | (n=177) | |  | (n=187) | |
|  |  | OR | 95% CI |  | OR | 95% CI |  | OR | 95% CI |  | OR | 95% CI |
| ***step 1*** |  |  |  |  |  |  |  |  |  |  |  |  |
| **Intention** |  | 5.60 | (4.10,7.64) |  | 2.34 | (1.20,4.54) |  | 3.14 | (2.09,4.71) |  | 2.02 | (1.26,3.26) |
| ***step 2*** |  |  |  |  |  |  |  |  |  |  |  |  |
| **Subjective norms** |  |  |  |  |  |  |  |  |  |  | 4.20 | (1.59,11.08) |
| ***step 3*** |  |  |  |  |  |  |  |  |  |  |  |  |
| **Habit strength** |  |  |  |  | 2.70 | (1.34,5.46) |  |  |  |  |  |  |
| **Information processing** |  |  |  |  |  |  |  |  |  |  | 0.39 | (0.21,0.76) |
| **Childhood vaccination ¥** |  |  |  |  |  |  |  |  |  |  |  |  |
| Not all |  | 1 |  |  |  |  |  |  |  |  |  |  |
| All |  | 10.74 | (3.17,36.32) |  |  |  |  |  |  |  |  |  |
| **Multivariable model** |  | pseudo-R^2^ | |  | pseudo-R^2^ | |  | pseudo-R^2^ | |  | pseudo-R^2^ | |
| Step 1 |  | 0.50 |  |  | 0.21 |  |  | 0.19 |  |  | 0.19 |  |
| Step 1+2 |  |  |  |  |  |  |  |  |  |  | 0.22 |  |
| Step 1+2+3 |  | 0.53 |  |  | 0.31 |  |  |  |  |  | 0.28 |  |
| *All variables significantly associated with HPV vaccination uptake in imputed analyses were included in the multivariable model for complete case analyses. | | | | | | | | | | | | |
| **Abbrevations:** | | | | | | | | | | | | |
| pseudo-R^2^ = pseudo R squared, is a number that indicates how well the data fit a logistic regression model. | | | | | | | | | | | | |
| NL denotes participants with a Dutch ethnicity, SNA denotes participants with a Surinamese, Netherlands Antillean or Aruban ethnicity, MENA denotes participants with a Middle Eastern or North African ethnicity (including Turkish participants), Other denotes participants from all other ethnicities. | | | | | | | | | | | | |
| Interpretation of the OR of continuous determinants: If a determinant increases with one unit, we expect to see the odds to be HPV vaccinated (i.e. received 1 or 2 doses) to increase with the specified ratio for that determinant. For example, if among the Dutch intention increases with one unit, we expect to see the odds to be HPV vaccinated to increase 5.60 times.  Interpretation of the OR of categorical determinants: the odds to be vaccinated are x-times higher in the non-reference categories compared to the reference category. For example, in the Dutch group if the daughter received all childhood vaccinations the odds to also become HPV vaccinated (i.e. received 1 or 2 doses) is 10.74 times higher compared to when the daughter did not receive all childhood vaccinations. | | | | | | | | | | | | |

| **Table S6**. Complete case analyses: Odds ratios for the association between key determinants and vaccination uptake among Dutch parents/guardians, and interaction between ethnic group and these determinants. HPV vaccination acceptability study among parents/guardians, in Amsterdam, the Netherlands, 2014. | | | | | | | | | | | | |
| --- | --- | --- | --- | --- | --- | --- | --- | --- | --- | --- | --- | --- |
|  |  |  | **Interaction effect** | | | | | | | | | |
|  |  |  | SNA vs Nl | |  | MENA vs NL | |  | Other vs NL | |  | Overall |
|  | NL-OR |  | xOR** | *p*-value |  | xOR** | *p*-value |  | xOR** | *p*-value |  | *p*-value |
| **Intention** | 4.99 |  | 0.64 | *0.255* |  | 0.63 | *0.088* |  | 0.53 | ***0.023*** |  | *0.091* |
| **Subjective norms** | 3.80 |  | 0.20 | ***0.041*** |  | 0.20 | ***0.005*** |  | 0.82 | *0.774* |  | ***0.007*** |
| **Habit strength** | 1.33 |  | 1.57 | *0.266* |  | 0.42 | ***<0.001*** |  | 0.55 | ***0.030*** |  | ***<0.001*** |
| **Information processing** | 0.84 |  | 0.33 | *0.065* |  | 1.25 | *0.452* |  | 0.53 | *0.113* |  | ***0.037*** |
| **Childhood vaccination** |  |  |  |  |  |  |  |  |  |  |  |  |
| Not all |  |  |  |  |  |  |  |  |  |  |  |  |
| All | 8.92 |  | 0.63 | *0.710* |  | 0.61 | *0.610* |  | 0.28 | *0.152* |  | *0.560* |
| NL denotes participants with a Dutch ethnicity, SNA denotes participants with a Surinamese, Netherlands Antillean or Aruban ethnicity, MENA denotes participants with a Middle Eastern or North African ethnicity (including Turkish participants), Other denotes participants from all other ethnicities. | | | | | | | | | | | | |
| *p*-values <0.05 are indicated in bold | | | | | | | | | | | | |
| Analyses were executed on complete cases. This resulted in 640 participants in the NL group, 99 participants in the SNA group, 173 participants in the MENA group, and 186 participants in the Other group. | | | | | | | | | | | | |
| Interpretation of the OR of continuous determinants: If a determinant increases with one unit, we expect to see the odds to be HPV vaccinated to increase with the specified ratio for that determinant (i.e. received 1 or 2 doses). For example, if among the Dutch intention increases with one unit, we expect to see the odds to be HPV vaccinated to increase 4.99 times.  Interpretation of the OR of categorical determinants: the odds to be vaccinated are x-times higher in the non-reference categories compared to the reference category. For example, in the Dutch group if the daughter received all childhood vaccinations the odds to also become HPV vaccinated (i.e. received 1 or 2 doses) is 8.92 times higher compared to when the daughter did not receive all childhood vaccinations.  Interpretation of xOR: this is the odds ratio of the interaction factor. It indicates the factor with which the OR of the Dutch group should be multiplied to obtain the odds ratio for that determinants in that particular non-Dutch group. For example, the xOR for subjective norm in the MENA group is 0.20. In order to obtain the odds ratio for the MENA-group for the effect of one step increase in the intention scale on vaccination uptake, one multiplies the OR of the Dutch group (3.80) with this xOR (0.20), resulting in an OR of 0.76. So the effect of subjective norm on vaccination uptake is significantly (*p*=0.005) less strong than in the Dutch group. | | | | | | | | | | | | |

| **Table S7**. HPV vaccination intention: multivariable linear regression analyses of complete cases of socio-demographic, social-psychological and other factors. HPV vaccination acceptability study among parents/guardians, in Amsterdam, the Netherlands, 2014. | | | | | | | | | | | | |
| --- | --- | --- | --- | --- | --- | --- | --- | --- | --- | --- | --- | --- |
|  |  | **Multivariable*** | | | | | | | | | | |
|  |  | **NL** | | **SNA** | | | **MENA** | | |  | **Other** | |
|  |  | (n=581) | |  | (n=110) | |  | (n=212) | |  | (n=174) | |
|  |  | ß | 95% CI |  | ß | 95% CI |  | ß | 95% CI |  | ß | 95% CI |
| *step 1* |  |  |  |  |  |  |  |  |  |  |  |  |
| **Attitude** |  | 0.65 | (0.56,0.75) |  | 0.66 | (0.43,0.88) |  | 1.02 | (0.87,1.16) |  | 0.68 | (0.52,0.83) |
| **Beliefs** |  | 0.34 | (0.21,0.48) |  | 0.24 | (0.01,0.48) |  |  |  |  | 0.34 | (0.14,0.55) |
| **Risk perception when not vaccinating** |  |  |  |  |  |  |  | 0.12 | (0.02,0.22) |  |  |  |
| **Relative effectiveness** |  | 0.17 | (0.05,0.30) |  |  |  |  |  |  |  |  |  |
| **Subjective norms** |  | 0.48 | (0.33,0.63) |  |  |  |  | 0.27 | (0.08,0.45) |  |  |  |
| **Descriptive norms** |  |  |  |  | 0.13 | (0.01,0.25) |  | 0.12 | (0.02,0.23) |  | 0.12 | (0.03,0.20) |
| *step 2* |  |  |  |  |  |  |  |  |  |  |  |  |
| **Ambivalence towards the decision** |  | -0.06 | (-0.12,-0.01) |  |  |  |  |  |  |  |  |  |
| **Information processing** |  | -0.06 | (-0.13,-0.00) |  |  |  |  |  |  |  |  |  |
| **Evaluation of the HPV information** |  | 0.22 | (0.13,0.31) |  | 0.37 | (0.16,0.57) |  |  |  |  | 0.19 | (0.06,0.32) |
| **Past experience with vaccinating older daughter against HPV** | | | |  |  |  |  |  |  |  |  |  |
| Older daughter not |  | REF |  |  |  |  |  |  |  |  | REF |  |
| Older daughter partially/fully vaccinated |  | 1.04 | (0.73,1.36) |  |  |  |  |  |  |  | 1.37 | (0.58,2.15) |
| No older daughter |  | 0.89 | (0.59,1.18) |  |  |  |  |  |  |  | 1.20 | (0.43,1.96) |
| **Past experience of someone close or him/herself with (prestage of) cervical cancer** | | | | | | |  |  |  |  |  |  |
| No |  |  |  |  |  |  |  |  |  |  | REF |  |
| Yes |  |  |  |  |  |  |  |  |  |  | 0.16 | (-0.00,0.32) |
| **Education** |  |  |  |  |  |  |  |  |  |  |  |  |
| Low |  | REF |  |  |  |  |  |  |  |  |  |  |
| Intermediate |  | 0.16 | (0.04,0.27) |  |  |  |  |  |  |  |  |  |
| High |  | 0.14 | (0.02,0.27) |  |  |  |  |  |  |  |  |  |
| **Religion** |  |  |  |  |  |  |  |  |  |  |  |  |
| No religion |  | REF |  |  |  |  |  |  |  |  |  |  |
| Religious |  | -0.18 | (-0.30,-0.07) |  |  |  |  |  |  |  |  |  |
| **Multivariable model** |  | **R^2^** |  |  | **R^2^** |  |  | **R^2^** |  |  | **R^2^** |  |
| Step 1 |  | 0.73 |  |  | 0.59 |  |  | 0.73 |  |  | 0.63 |  |
| Step 1+2 |  | 0.75 |  |  | 0.59 |  |  |  |  |  | 0.70 |  |
| *All variables significantly associated with HPV vaccination uptake in imputed analyses were included in the multivariable model for complete case analyses. | | | | | | | | | | | | |
| **Abbrevations:** | | | | | | | | | | | | |
| R^2^ = R squared, is a number that indicates how well the data fit a linear regression model. | | | | | | | | | | | | |
| NL denotes participants with a Dutch ethnicity, SNA denotes participants with a Surinamese, Netherlands Antillean or Aruban ethnicity, MENA denotes participants with a Middle Eastern or North African ethnicity (including Turkish participants), Other denotes participants from all other ethnicities. | | | | | | | | | | | | |
| Interpretation of the coefficient of continuous determinants: if a determinant increases with one unit we expect to see an increase in intention with the coefficient specified for that determinant. For example, among the Dutch, if attitude increases with one unit, we expect intention to increase with 0.65.  Interpretation of the coefficient of categorical determinants: the intention to be vaccinated is ß higher or lower in the non-reference category when compared to the reference category. For example, overall, Dutch participants that are highly educated have an intention that is 0.14 higher (on the scale of -2 to +2) when compared to those participants with a low education. | | | | | | | | | | | | |

| **Table S8**. Complete case analyses: Regression coefficient for the association between key determinants and vaccination intention among Dutch parents/guardians, and interaction between ethnic group and these determinants. HPV vaccination acceptability study among parents/guardians, in Amsterdam, the Netherlands, 2014. | | | | | | | | | | | | | |
| --- | --- | --- | --- | --- | --- | --- | --- | --- | --- | --- | --- | --- | --- |
|  |  |  |  | **Interaction effect** | | | | | | | | | |
|  |  |  |  | SNA vs Nl | |  | MENA vs NL | |  | Other vs NL | |  | Overall |
|  |  | NL-ß |  | ∆ß** | *p*-value |  | ∆ß | *p*-value |  | ∆ß | *p*-value |  | *p*-value |
| **Attitude** |  | 0.77 |  | -0.12 | *0.220* |  | 0.14 | *0.030* |  | -0.10 | *0.149* |  | ***0.015*** |
| **Beliefs** |  | 0.36 |  | -0.21 | *0.080* |  | -0.07 | *0.472* |  | -0.10 | *0.317* |  | *0.302* |
| **Risk perception when not vaccinating** |  | 0.06 |  | -0.14 | ***0.035*** |  | 0.02 | *0.760* |  | -0.05 | *0.399* |  | *0.137* |
| **Relative effectiveness** |  | 0.16 |  | -0.31 | ***0.031*** |  | -0.14 | *0.184* |  | -0.26 | ***0.017*** |  | *0.200* |
| **Subjective norms** |  | 0.42 |  | -0.46 | ***0.001*** |  | -0.03 | *0.749* |  | -0.26 | ***0.023*** |  | ***0.001*** |
| **Descriptive norms** |  | 0.08 |  | -0.11 | *0.167* |  | 0.01 | *0.821* |  | -0.06 | *0.351* |  | *0.369* |
| **Ambivalence towards the decision** |  | -0.06 |  | -0.03 | *0.584* |  | -0.02 | *0.726* |  | 0.01 | *0.856* |  | *0.922* |
| **Information processing** |  | -0.08 |  | 0.00 | *0.963* |  | 0.12 | ***0.035*** |  | 0.00 | *0.967* |  | *0.176* |
| **Evaluation of the HPV information** |  | 0.23 |  | -0.06 | *0.601* |  | -0.14 | ***0.046*** |  | -0.11 | *0.141* |  | *0.172* |
| **Past experience with vaccinating older daughter against HPV** | | | | |  |  |  |  |  |  |  |  |  |
| Older daughter not |  | REF |  |  |  |  |  |  |  |  |  |  | ***0.012*** |
| Older daughter partially/fully vaccinated |  | 1.06 |  | -0.66 | ***0.035*** |  | -0.61 | ***0.015*** |  | -0.09 | *0.843* |  |  |
| No older daughter |  | 0.89 |  | -0.72 | ***0.009*** |  | -0.76 | ***<0.001*** |  | -0.02 | *0.958* |  |  |
| **Past experience of someone close or him/herself with (prestage of) cervical cancer** | | | | | | | |  |  |  |  |  |  |
| No |  | REF |  |  |  |  |  |  |  |  |  |  |  |
| Yes |  | 0.07 |  | 0.08 | *0.581* |  | -0.09 | *0.480* |  | 0.05 | *0.672* |  | *0.763* |
| **Education** |  |  |  |  |  |  |  |  |  |  |  |  |  |
| Low |  | REF |  |  |  |  |  |  |  |  |  |  | *0.070* |
| Intermediate |  | 0.16 |  | 0.10 | *0.488* |  | -0.04 | *0.736* |  | -0.31 | ***0.018*** |  |  |
| High |  | 0.16 |  | 0.25 | *0.240* |  | 0.01 | *0.945* |  | -0.20 | *0.118* |  |  |
| **Religion** |  |  |  |  |  |  |  |  |  |  |  |  |  |
| No religion |  | REF |  |  |  |  |  |  |  |  |  |  | *0.084* |
| Religious |  | -0.19 |  | 0.07 | *0.662* |  | -0.04 | *0.837* |  | 0.27 | ***0.012*** |  |  |
| *p*-values <0.05 are indicated in bold | | | | | | | | | | | | | |
| Analyses were executed on complete cases. This resulted in 569 participants in the NL group, 99 participants in the SNA group, 154 participants in the MENA group, and 161 participants in the Other group. | | | | | | | | | | | | | |
| Interpretation of the coefficient of continuous determinants: if a determinant increases with one unit we expect to see an increase in intention with the coefficient specified for that determinant. For example, among the Dutch, if attitude increases with one unit, we expect intention to increase with 0.77.  Interpretation of the coefficient of categorical determinants: the intention to be vaccinated is ß higher or lower in the non-reference category when compared to the reference category. For example, overall, Dutch participants that are highly educated have an intention that is 0.16 higher (on the scale of -2 to +2) when compared to those participants with a low education.  Interpretation of ∆ß: Delta Beta is the difference in the beta in one of the non-Dutch groups when compared to the NL-group. For example, for the variable Subjective Norms, the ∆ß in the SNA-group is -0.46, indicating that the slope is 0.46 lower in the SNA-group compared to the NL-group; this is a significant effect (*p*=0.001). | | | | | | | | | | | | | |


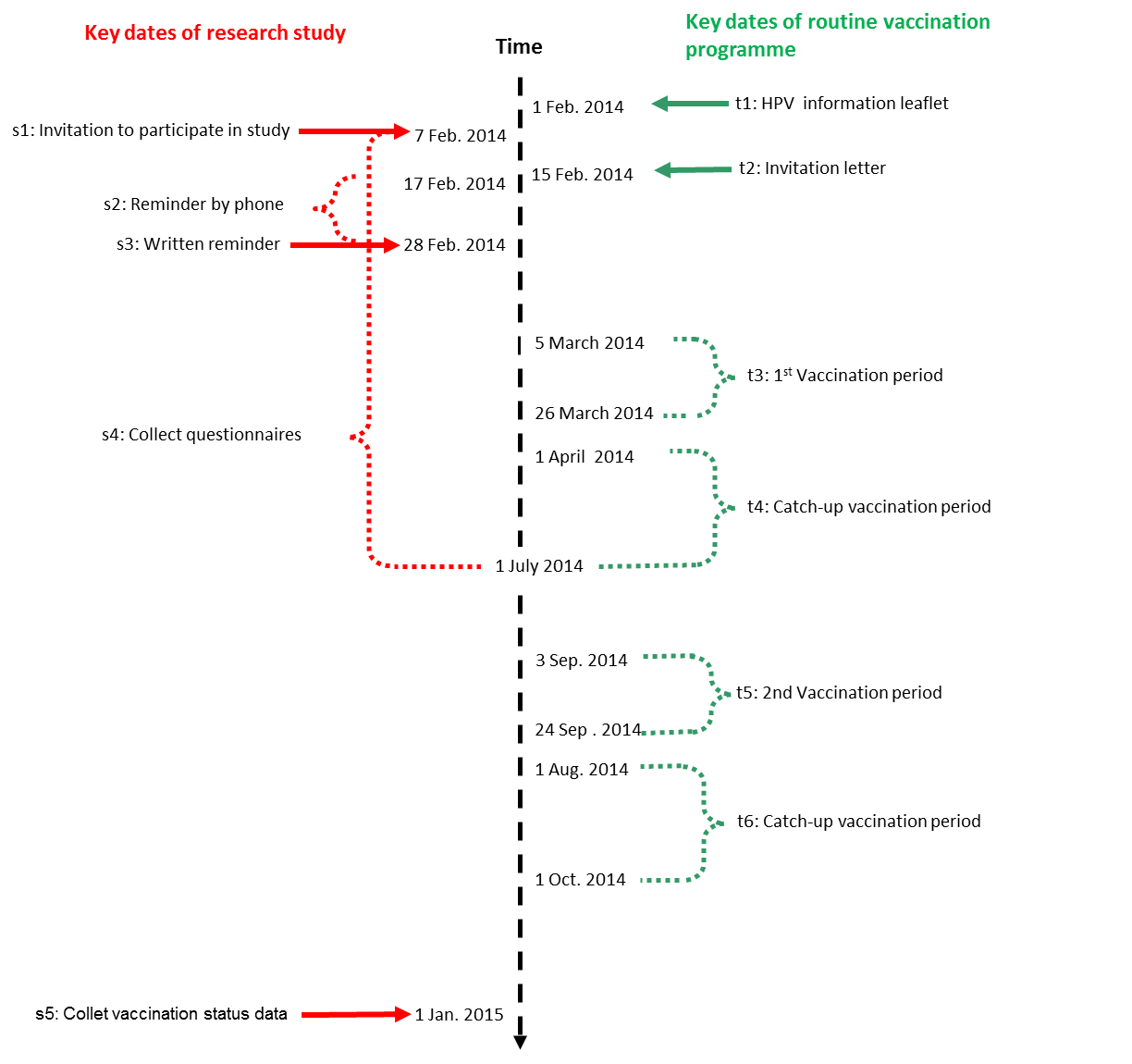


**Figure S1**. Schematic representation of key dates and periods: HPV vaccination acceptability study, Amsterdam 2014 The vertical line indicates the time. Texts at the arrows indicate each specific time point or period. An arrow indicates a specific time point and a brace indicates a specific period of time. On the right side of the timeline are dates and periods relating to the routine vaccination program and on the left side of the timeline are dates and periods relating to the research project. In short, girls are invited for the HPV vaccination in the year that they turn 13 years of age. At **t1** information and at **t2** an invitation letter (with time and place of vaccination) regarding the HPV vaccination was sent by the National Immunization Program (NIP) of the National Institute for Public Health and the Environment (RIVM) in the Netherlands. In Amsterdam, girls were invited to get the first dose of HPV vaccination on one of the seven vaccination-days during a one month period (**t3)** according to the zip code of the girl’s residence. During period **t4** a catch-up was organized for those who missed the vaccination during t3. During the period indicated with **t5** the second dose of the HPV vaccination was offered, and during the period indicated with **t6** a catch-up for the second HPV vaccination was offered. At **s1** the invitation for this study was sent to the parents/guardians. Starting 10 days after **s1**, during a period of two weeks (**s2**), parents/guardians who had not responded yet were called by phone to remind them about the study and assist them where needed. Three weeks after **s1**, parents/guardians who had not returned the questionnaire received a written reminder (**s3**). During a period of 5 months questionnaires were received, systematically stored and entered in the study database (**s4**). At **s5** the vaccination status was downloaded from the data registry of an electronic national registry of the NIP, and subsequently merged with the questionnaire data.


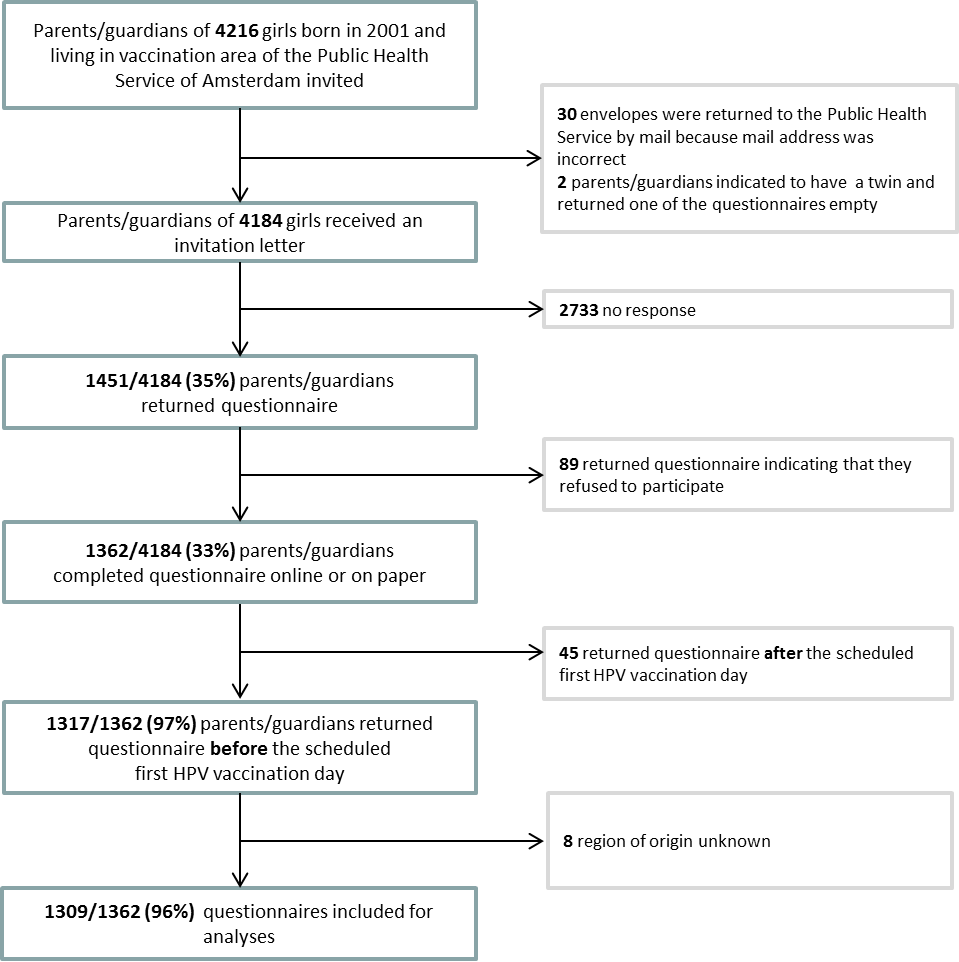


**Figure S2**. Flow diagram of the recruitment and response of parents/guardians and their daughters of the HPV vaccination acceptability study, Amsterdam 2014.


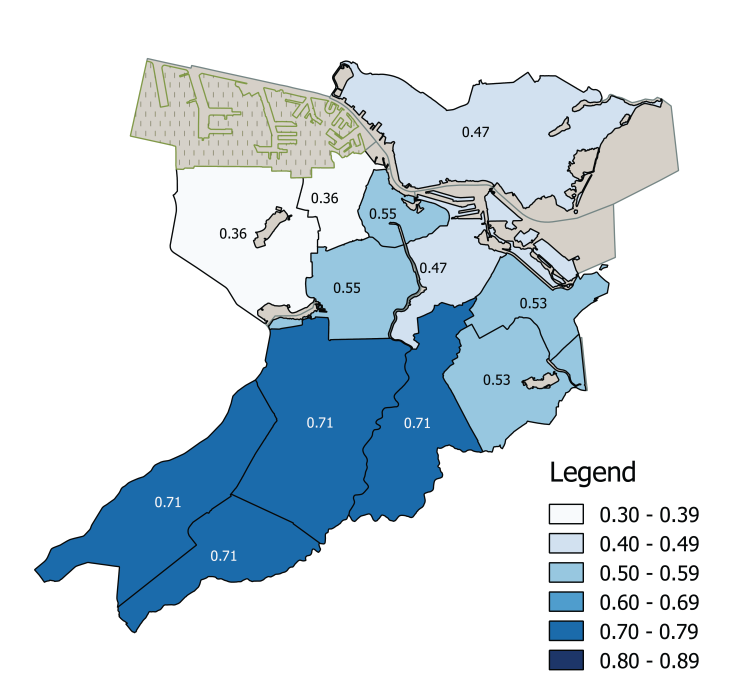

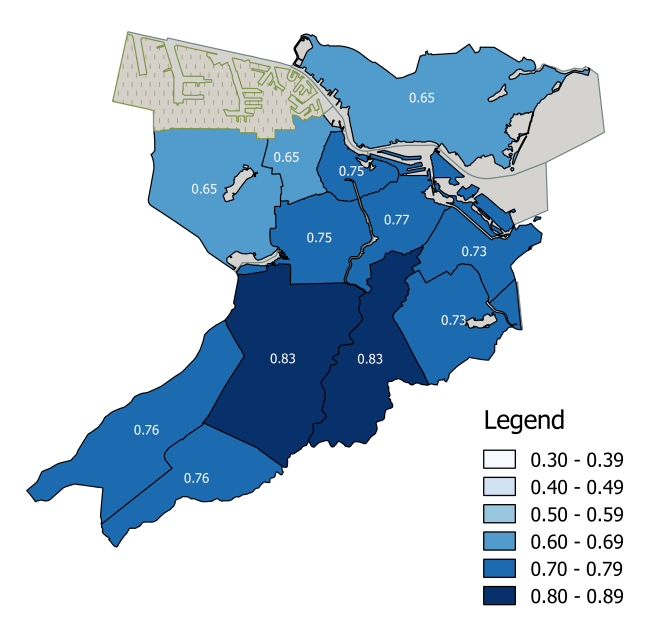


**Figure S3**. Proportion of girls that received two HPV vaccine doses by neighborhood in the health district of the Public Health Service of Amsterdam. Each neighborhood has one location where the HPV vaccination is given. In the left figure we present the HPV vaccination uptake for all girls invited for the HPV vaccination in 2014 (total HPV vaccination uptake 51%) and in the right figure we present the HPV vaccination uptake of all girls participating in this study (n=1,309) (total HPV vaccination uptake 73%) .
